# Multifunctional magnetic nanoparticles elicit anti-tumor immunity in a mouse melanoma model

**DOI:** 10.1016/j.mtbio.2023.100817

**Published:** 2023-09-24

**Authors:** Nuria Lafuente-Gómez, Irene de Lázaro, Mónica Dhanjani, David García-Soriano, Miguel C. Sobral, Gorka Salas, David J. Mooney, Álvaro Somoza

**Affiliations:** a Instituto Madrileño de Estudios Avanzados en Nanociencia (IMDEA Nanociencia), Madrid, 28049, Spain; b Harvard John A. Paulson School of Engineering and Applied Sciences, Harvard University, Cambridge, MA, 02138, USA; c Wyss Institute for Biologically Inspired Engineering, Harvard University, Boston, MA, 02115, USA; d Immunology Service, Hospital Universitario de la Princesa, Instituto Investigación Sanitaria Princesa, Madrid, 28006, Spain; e Department of Biomedical Engineering, NYU Tandon School of Engineering, New York University, New York, NY, 10010, USA; f NYU Cardiovascular Research Center, Division of Cardiology, Department of Medicine, NYU Grossman School of Medicine, New York University, New York, NY, 10010, USA; g Unidad de Nanobiotecnología Asociada al Centro Nacional de Biotecnología (CNB-CSIC), Madrid, 28049, Spain

**Keywords:** Magnetic nanoparticles, Nanomedicine, Cancer immunotherapy, Cancer vaccine

## Abstract

Immunotherapy has emerged as a promising strategy to eradicate cancer cells. Particularly, the development of cancer vaccines to induce a potent and sustained antigen-specific T cell response has become a center of attention. Herein, we describe a novel immunotherapy based on magnetic nanoparticles (MNP) covalently modified with the OVA_254-267_ antigen and a CpG oligonucleotide *via* disulfide bonds. The MNP-CpG-COVA significantly enhances dendritic cell activation and CD8^+^ T cell antitumoral response against B16-OVA melanoma cells *in vitro*. Notably, the immune response induced by the covalently modified MNP is more potent and sustained over time than that triggered by the free components, highlighting the advantage of nanoformulations in immunotherapies. What is more, the nanoparticles are stable in the blood after *in vivo* administration and induce potent levels of systemic tumor-specific effector CD8 + T cells. Overall, our findings highlight the potential of covalently functionalized MNP to induce robust immune responses against mouse melanoma.

## Graphical abstract

**Figure:**
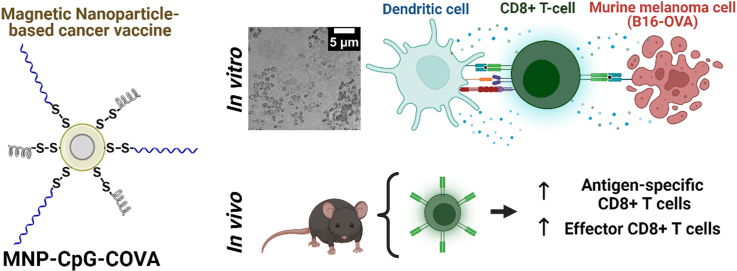

## 1 Introduction

Cancer remains one of the largest public health problems worldwide [1]. Fortunately, remarkable progress in cancer treatments has been achieved in recent years. Among them, immunotherapy, which consists of directing the patient's immune system against the tumor, has provided outstanding results [2]. One strategy to induce antitumor immunity is through cancer vaccines that deliver tumor antigens combined with adjuvants to antigen-presenting cells (APCs), (e.g., dendritic cells, DCs), resulting in their activation, antigen presentation in secondary lymphoid organs, and induction of a potent and sustained antitumoral T cell response [3,4]. Nevertheless, to achieve such an ideal response, cancer vaccination must tackle some challenges that include: 1) efficiently delivering vaccine components to target cells, 2) preventing their premature degradation, and 3) avoiding off-target effects. Altogether, precise control of the immune system is needed to prevent autoimmunity and overcome the immunosuppressive tumor microenvironment [3,[5], [6], [7]].

In this regard, nanotechnology offers a wide variety of platforms that can be used as carriers of antigens and/or adjuvants to protect them from premature release, degradation, and to improve their physicochemical properties (e.g., solubility) and pharmacokinetic characteristics (e.g., rapid metabolism and excretion) [[8], [9], [10], [11]]. Additionally, nanoparticle-based vaccines are generally easily recognized and internalized by APCs because these are specialized in phagocytosing foreign materials [[12], [13], [14], [15], [16]]. Magnetic nanoparticles (MNP) have been explored for cancer immunotherapy for their high biocompatibility, intrinsic adjuvant properties, and ease of surface modification for controlled drug release applications [[17], [18], [19], [20]]. Moreover, they can be degraded and metabolized into ferritin that is incorporated into cellular metabolic routes, preventing any toxicity issues [[21], [22], [23]]. MNP can be easily obtained in large quantities and cost-effectively through coprecipitation synthesis using iron chloride salts, all while avoiding the generation or involvement of toxic intermediates or solvents This makes them ideal materials for large-scale industrial production [[24], [25], [26]]. In addition, the magnetic properties of these nanoparticles can be utilized to track their biodistribution using imaging techniques such as MRI [17,27,28]. As a result, MNP have found broad applications in the medical field, including the treatment of anemia and the development of diagnostic tools imaging, among other uses [29].

Herein, we developed a cancer immunotherapy based on MNP covalently modified with an antigen peptide (a cysteine-modified class I restricted epitope of ovalbumin, Cys-OVA_257-264_, COVA) and an oligonucleotide adjuvant (CpG ODN1826, an agonist of murine Toll-like receptor 9, TLR9 [30]) *via* disulfide bonds (MNP-CpG-COVA). It is worth remarking that previously described magnetic nanoparticles-based nanoformulations are mainly based on electrostatic interactions [[31], [32], [33]] where the active components could be released once the formulations are exposed to the biological media, leading to reduced effects. In our approach, the use of disulfide bonds to covalently conjugate the peptide and the oligonucleotide presents two major advantages, making the nanoformulation highly effective. Firstly, they ensure the stability of the formulation in the bloodstream because, unlike in intracellular media, the levels of glutathione in blood circulation and extracellular media are insufficient to break these bonds [[34], [35], [36]]. Secondly, antigen peptides conjugated *via* reducible bonds are much more efficiently processed and presented by DCs than those conjugated by their non-reducible counterparts [37,38]. Furthermore, the ease of functionalization inherent in our MNP-CpG-COVA system allows for customization based on patient-specific characteristics. In this study, we first examined the biocompatibility of MNP and their ability to induce an antigen-specific immune response and tumor killing *in vitro.* Particularly, the maturation of dendritic cells and the activation of antigen-specific cytotoxic T cells against mouse melanoma cells were evaluated. Next, we investigated the immune response triggered by our cancer vaccine in a murine model. Overall, we thoroughly explored the potential use of modified MNP in cancer immunotherapy, demonstrating its ability to induce antigen-specific effector T cell responses both *in vitro* and *in vivo*. It is worth highlighting that the system can be produced in a straightforward manner and can be easily personalized. Consequently, this approach paves the way for groundbreaking advancements in immunotherapy and the enhancement of cancer treatment strategies tailored to individual patients.

## 2 Materials and methods

### 2.1 Synthesis and characterization of magnetic nanoparticles (MNP)

Magnetic nanoparticles composed of maghemite (γ-Fe_2_O_3_) cores were synthesized by the coprecipitation method followed by acid treatment, as described elsewhere [39,40], and coated with carboxymethyldextran according to previous protocols [18,41]. The details of the procedure can be found in the Supplementary Material. Inductively coupled plasma optical emission spectrometry (ICP-OES) was used to determine the iron concentration. Simultaneous thermogravimetric/differential thermal analyses (TGA/DTA) were done in a TA Instruments TGA 500, with a heating rate of 10 °C min^-1^, in air atmosphere from room temperature to 800 °C to determine the percentage of weight loss, thus calculate the proportion between the organic (carboxymethyldextran) and inorganic (γ-Fe_2_O_3_ cores) layers.

For their characterization, the size and shape of the nanoparticles were examined by Transmission Electron Microscopy (JEOL JEM 1010) at Facultad de Medicina, Universidad Autónoma de Madrid. One drop of a diluted dispersion of MNP was added to a carbon-coated copper grid and left to dry overnight prior to visualization. The hydrodynamic diameter and ζ potential were obtained in a Zetasizer (DLS, NanoZS device, Malvern Instruments) in aqueous dispersions at pH 7.4 of MNP (0.05 mg Fe·mL^-1^, n = 3) at 25 °C.

For the detection of endotoxins, ToxinSensor™ Gel Clot Endotoxin Assay Kit (GenScript) was employed following manufacturer's instructions (limit of detection 0.25 EU·mL^-1^). The positive control was composed of an endotoxin standard dissolved to a final concentration of 0.25 EU·mL^-1^, and the negative control was composed of miliQ water (Merck Millipore). The MNP samples were tested at several concentrations (53.75, 2, 0.5 and 0.05 mg Fe·mL^-1^). In the presence of endotoxins, gelation occurred; in the absence of endotoxin, gelation did not occur.

### 2.2 Functionalization of MNP with polyTCy5, CpG and OVA_257-264_*via* disulfide bonds (MNP-PolyTCy5, MNP-CpG, MNP-COVA, MNP-CpG-COVA)

For the functionalization of MNP, a similar described procedure was employed [18]. Briefly, MNP (1 mL, 2 mg Fe·mL^-1^) were incubated overnight with 1-ethyl-3-(3-dimethylaminopropyl)-carbodiimide hydrochloride (EDC, Sigma Aldrich; 20 μL 120 mM, 600 μmol per g Fe) and N-hydroxysuccinimide (NHS, Sigma Aldrich; 20 μL 60 mM, 300 μmol per g Fe). Next, the nanoparticles were washed by three cycles of centrifugation and redispersion (12,000 g, 1 h). Then, a solution of cysteamine hydrochloride (CistHCl, Sigma Aldrich; 20 μL 40 mM, 200 μmol per g Fe) previously neutralized with NaOH (Sigma Aldrich, 20 μL 40 mM, 200 μmol per g Fe) was added, and the mixture was stirred overnight. Later, the nanoparticles were washed as mentioned before and mixed overnight with aldrithiol (Sigma Aldrich, 200 μL 40 mM 500 μM in dimethylformamide, DMF, 50 μmol per g Fe). The obtained nanoparticles (MNPssPyr) were washed and the release of 2-pyridinethione (Pyr) in the supernatant (*λ*_343 nm_, ε = 8080 L·mol^-1^·L^-1^; Synergy Neo2 Plate Reader (BioTek Instruments) was employed to assess the yield of the reaction (100%, 50 μmol Pyr per g Fe). For the functionalization with oligonucleotides (PolyCy5 or CpG, both obtained from IDT, Table S1), they were previously deprotected with Tris(2-carboxyethyl)phosphine hydrochloride (TCEP, Sigma Aldrich) for 2 h and purified by NAP-5 column (Fisher Scientific) following manufacturer's instructions. For the functionalization with the peptide OVA_257-264_, its sequence was modified with a cysteine in the N-terminus (COVA, Peptide 2.0 Inc, Table S1). The oligonucleotides (CpG or PolyTCy5) and/or the peptide (COVA) were added to the dispersion of MNPssPyr (1 mL, 2 mg Fe·mL^-1^) to a final concentration of 2.5 μmol per g Fe (100 μL 50 μM in water) and incubated overnight. The following day, the samples were centrifuged (8000 g, 20 min) and resuspended in Mili-Q water. The amount of CpG, PolyTCy5, and COVA incorporated was determined by the quantification of 2-pyridinethione (Pyr) released, as previously mentioned. Additionally, Micro BCA™ Protein Assay Kit (Thermofisher) was employed to quantify the amount of COVA in the supernatant, enabling calculation of the amount of peptide attached to the nanoparticles. The final amount of attached COVA and CpG was 2.5 μmol/g Fe each.

### 2.3 Animal studies

C57BL/6 and C57BL/6-Tg(TcraTcrb)1100Mjb/J (OT-I) mice were purchased from Jackson Laboratories and were under Harvard University Faculty of Arts and Sciences (FAS) Institutional Animal Care. All procedures were performed according to Harvard University's Institutional Animal Care and Use Committee (IACUC) guidelines under protocol 24-16.

### 2.4 Hemocompatibility studies with mouse red blood cells

To assess the hemocompatibility of the MNP, the rupturing (lysis) of red blood cells (RBC) upon incubation with MNP was analyzed employing the release of hemoglobin as a marker, as previously described [18,42,43]. Briefly, the release of hemoglobin after the incubation of RBCs and MNP was quantified by absorbance (*λ*_540 nm_) in a Synergy Neo2 Plate Reader (BioTek Instruments). The details of the procedure are found in the Supplementary Material.

### 2.5 Isolation, culture and differentiation of bone marrow derived dendritic cells (BMDC)

Primary BMDC were isolated from female C57BL/6 mice aged between 6 and 10 weeks old (Jackson Laboratories, USA) using standard methods [44]. In brief, bone marrow was collected from the femur and tibia bones and the single-cell suspensions were cultured in RPMI (ThermoFisher) supplemented with 10% heat-inactivated fetal bovine serum (HI-FBS, Sigma Aldrich), 1% penicillin-streptomycin (Fisher Scientific), 50 μM β-mercaptoethanol (Sigma Aldrich) and 20 ng mL^-1^ murine Granulocyte-Macrophage Colony-Stimulating factor (GM-CSF, Prepotech). Non-adherent and loosely adherent cells after 7 days of differentiation were collected and used for the studies.

### 2.6 Cytocompatibility studies with BMDC

- *Alamar Blue cell viability assay*. BMDC (40,000 cells per well) were seeded in 96-well tissue culture treated plates (VWR) and incubated with the treatments for 4, 24 and 48 h at 37 °C, 5% CO_2_ (n = 6). Then, cells were washed twice with PBS and alamarBlue HS cell Viability Reagent (ThermoFisher) was added according to manufacturer's instructions. The fluorescence was measured (*λ*_ex_ 560 nm, *λ*_em_ 590 nm) in a Synergy Neo2 Plate Reader (BioTek Instruments). The measurements were processed using equation (2), wherein the positive control corresponds with untreated cells, and the negative control is a solution of alamarBlue HS cell Viability Reagent as employed in the assay without cells.

(2) $$\%\;Cell\;viability=\frac{Sample\;data-Negative\;Control}{Positive\;Control-Negative\;Control}\;x\;100$$

- *Annexin V/7-amino-actinomycin D (7-AAD) assay for necrosis/apoptosis detection.* BMDC (250,000 cells per well) were seeded in 24-well tissue culture treated plates (VWR) and incubated with the treatments for 4, 24, and 48 h at 37 °C, 5% CO_2_. Cells were then washed twice with PBS, detached from the well plate by scraping, and washed twice in FACS buffer (PBS, 1% BSA). Then, APC- Annexin V/7-AAD kit (Biolegend) was employed following the manufacturer's instructions, and the samples were analyzed using the Aurora Spectral Cytometer (Cytek). Unstained cells, Annexin V stained cells and 7-AAD stained cells were recorded for the spectral unmixing performed using the SpectroFlo V2.2 software (Cytek). Gates were validated with the single stained controls, and the results were processed using FCS Express 7 (De Novo Software). The populations were established as follows: Annexin V -/7-ADD – were considered live cells, Annexin V +/7-ADD – early apoptotic cells, Annexin V +/7-ADD + late apoptotic/necrotic cells, and Annexin V -/7-ADD + dead cells due to the harvesting procedure.
- *Cell cycle analysis.* BMDC (500,000 cells per well) were seeded in 12-well tissue culture treated plates (VWR) and incubated with the treatments for 4, 24, and 48 h at 37 °C, 5% CO_2_. Then, cells were washed twice with PBS, detached from the well plate by scraping, and fixed in cold ethanol 70% for 15 min on ice. Later, the ethanol was removed by three cycles of centrifugation and redispersion in PBS (350 g, 5 min). Each sample was resuspended in 500 μL of PBS and treated with 10 μg RNAase A (Sigma Aldrich) and 20 μg propidium iodide (Sigma Aldrich). Then, samples were analyzed using the Aurora Spectral Cytometer (Cytek). Unstained cells and cells stained with PI were recorded for the spectral unmixing performed by using SpectroFlo V2.2 software (Cytek). The results were processed using FCS Express 7 (De Novo Software).

### 2.7 Internalization studies in BMDC

- *Colorimetric ferrozine assay* [45]. BMDC (250,000 cells per well) were seeded in flat bottom 24-well tissue culture treated plates (VWR) and incubated with the treatments for 4, 24 and 48 h at 37 °C, 5% CO_2_ (n = 3). Then, cells were washed twice with PBS, detached from the well plate by scraping, and digested in 100 μL of NaOH 50 mM (Sigma Aldrich) for 1 h at 60 °C. Then, the ferrozine assay was performed as described in Supplementary Material.
- *Prussian Blue staining* [46]. BMDC (60,000 cells per well) were seeded in 8-well glass Millicell EZ slide (Merk) and incubated with the treatments for 4, 24 and 48 h (n = 6). Then, cells were washed twice with PBS and fixed in ice-cold methanol for 5 min. Next, the cells were stained as described in Prussian Blue section of Supplementary Material.
- *TEM images.* BMDC (800,000 cells per well) were seeded in 6-well tissue culture treated plates (VWR) and incubated with the treatments for 48 h at 37 °C, 5% CO_2_. Then, cells were washed twice with PBS, detached from the plate by scraping and fixed for 1 h at room temperature in 200 μL of glutaraldehyde 2.5% and formaldehyde 2% in Sodium cacodylate buffer 0.1 M pH 7.4 (LADD Research Industries). Cells were kept refrigerated until further processing at the Electron Microscopy Facility of Harvard Medical School. The samples were visualized in a JEOL1200 EX.
- *Confocal images.* BMDC (60,000 cells per well) were seeded in 8-well glass Millicell EZ slide (Merk) and incubated with the treatments for 4, 24, and 48 h at 37 °C, 5% CO_2_. Then, cells were washed twice with PBS and fixed with a solution of paraformaldehyde 4% in PBS for 15 min at room temperature. Next, a solution of Triton X-100 0.1% in PBS was added for 15 min to permeabilize the cells. Actin filaments were stained upon incubation with 1 X phalloidin conjugated with AlexaFluor488 (ThermoFisher) for 30 min, and the nucleus DNA was stained with DAPI 10 μg·mL^-1^ after 15 min of incubation. Samples were visualized in a ZEISS LSM880 (Carl Zeiss AG).

### 2.8 *In vitro* release studies

The selectivity of the disulfide bond to be broken under highly reducing conditions was tested *in vitro* using MNP-PolyTCy5 as a model. Briefly, MNP-PolyTCy5 (2 mg Fe·mL^-1^) were incubated in PBS at 37 °C (pH 7.4, 1 mL) with two different concentrations of the reducing agent 1.4-dithiothreitol (DTT, Sigma Aldrich). Particularly, 1 μM DTT mimicked the extracellular and blood conditions [34] whereas 1 mM DTT imitated the intracellular reducing environment thanks to the presence of gamma interferon-inducible lysosomal thiolreductase (GILT) in antigen-presenting cells [47,48], such as dendritic cells. The amount of PolyTCy5 released was determined by measuring the absorbance of Cy5 (*λ*_646 nm_, ε_646 nm_ = 250,000 L·mol^-1^·cm^-1^) in a Synergy H4 microplate reader (Agilent Biotek).

### 2.9 Evaluation of BMDC activation state

BMDC (250,000 cells per well) were seeded in 24-well tissue culture treated plates (VWR) and incubated with the treatments for 24 or 48 h at 37 °C, 5% CO_2_. Then, cells were harvested and incubated in 50 μL of FACS buffer (PBS, 1% BSA) containing 5 μg·mL^-1^ antimouse CD16/CD32 monoclonal antibody (ThermoFisher) for 5 min at 4 °C. Next, 50 μL of staining solution in FACS buffer (CD11c-SB436, I-A/I-E-BV480, CD40-FITC and CD86-BUV395; 1 μg·mL^-1^ each) was added and incubated for 30 min at 4 °C. Cells were washed three times with FACS buffer and analyzed using the Aurora spectral cytometer (Cytek). Unstained cells and single stained controls of cells treated with LPS 5 ng·mL^-1^ for 24 or 48 h were used to unmix the different channels with SpectroFlo V2.2 software. The gating strategy can be found in the Supplementary Material (Fig. S13). The results were analyzed using FCS Express 7 (De Novo Software).

### 2.10 BMDC antigen presentation assay

BMDC (500,000 cells per well) were seeded in 12-well tissue culture treated plates (VWR) and incubated with the treatments for 24 or 48 h at 37 °C, 5% CO_2_. Then, cells were harvested and incubated in 50 μL of FACS buffer (PBS, 1% BSA) containing 5 μg·mL^-1^ antimouse CD16/CD32 monoclonal antibody (ThermoFisher) for 5 min at 4 °C. Then, 50 μL of FACS buffer containing antimouse CD11c-SB436 and antimouse H-2Kb/SIINFEKL- PE/Cy7 (1 μg·mL^-1^ each) were added and the samples were incubated for 30 min at 4 °C. Next, they were washed three times with FACS buffer and analyzed using the Aurora spectral cytometer (Cytek). Unstained cells and single stained controls of cells treated with COVA were used to unmix the different channels with SpectroFlo V2.2 software. For the gating strategy, CD11c + viable cells were selected to evaluate the % H-2Kb/SIINFEKL + cells within that population. The data was analyzed using FCS Express 7 (De Novo Software).

### 2.11 Isolation and culture of CD8^+^ T-cells

CD8^+^ T-cells were isolated from female C57BL/6-Tg(TcraTcrb)1100Mjb/J (OT-I) and C57BL/6 mice spleens aged between 6 and 10 weeks old (Jackson Laboratories). The isolated spleens were disrupted, erythrocyte lysis buffer was used (Red Blood Cell Lysis Buffer, Thermofisher) and CD8^+^ T cell isolation kit for mouse (Miltenyi Biotech) was employed to separate the desired T cells. The T cell culture media used for their incubation was composed of RPMI 1640 (ThermoFisher) supplemented with 10% Heat Inactivated Fetal Bovine Serum (HI-FBS, Sigma Aldrich), 2 mM L-glutamine (Fisher), 100 U/L Penicillin/Streptomicin (ThermoFisher), 5 mM sodium pyruvate (Lonza), 1 X non-essential aminoacids (Thermofisher), 50 μM β-mercaptoethanol (Sigma Aldrich) and 24 ng mouse IL-2·mL^-1^ (Preprotech).

### 2.12 *Ex vivo* BMDC:CD8+ T cell coculture

BMDC (40,000 cells per well) were seeded in U-bottom 96-well tissue culture treated plates (VWR) and incubated with the treatments for 6 h at 37 °C, 5% CO_2_. Next, CD8^+^ T cells from OT-I mice (40,000 cells per well) were added to the plate and incubated for 48 h. Then, the proliferation and activation of T cells were evaluated.

- *CD8*^*+*^*T cell expansion study.* Viable CD8^+^ T cells isolated from OT-I mice were diluted in prewarmed PBS to a density of 1 × 10^6^ cells·mL^-1^ and labeled with carboxyfluorescein diacetate succinimidyl ester (CFSE) 1 μM (CellTrace CFSE Cell Proliferation Kit, Thermofisher) upon incubation for 20 min at 37 °C, 5% CO_2_. Next, cells were extensively washed to remove the excess of CFSE and added to the wells (40,000 CFSE labeled T cells) with BMDC. After 48 h of coculture at 37 °C, 5% CO_2_, cells were harvested, washed twice FACS buffer (PBS, 1% BSA), and resuspended in 50 μL of FACS buffer containing 5 μg·mL^-1^ antimouse CD16/CD32 monoclonal antibody (ThermoFisher) for 5 min at 4 °C. Then, 50 μL of FACS buffer containing antimouse CD3-PacificBlue (1 μg·mL^-1^) were added and incubated for 30 min at 4 °C. Next, samples were washed three Unstained and single stained cells treated with CpG and COVA were used as controls to unmix the different channels with SpectroFlo V2.2 software. The gating strategy is described in the Supplementary Material (Fig. S14). The data was evaluated using FCS Express 7 (De Novo Software). Results were expressed as % divided cells within CD3^+^ population and as proliferation index (the total number of divisions divided by the number of cells that went into division). These data were calculated using the Proliferation Statistics of FCS Express 7 (De Novo Software).
- *Intracellular Cytokine Staining of CD8*^*+*^*T-cells.* BMDC and CD8^+^ T-cells from OT-I mice were coincubated for 48 h as previously mentioned. Then, GolgiPlug (BD Biosciences; 0.2 μL per well) was added to the cells and incubated for 4 h at 37 °C, 5% CO_2_ to stop cytokine secretion. Next, cells were washed twice with PBS and stained with LIVE/DEAD™ Fixable Near-IR (ThermoFisher) according to manufacturer's instructions. Then, cells were resuspended in 50 μL of FACS buffer containing 5 μg·mL^-1^ antimouse CD16/CD32 monoclonal antibody (ThermoFisher) for 5 min at 4 °C. Then, 50 μL of FACS buffer containing antimouse CD3-PacificBlue (1 μg·mL^-1^) were added and incubated for 30 min at 4 °C. Samples were washed twice with FACS buffer, and then fixed and permeabilized with Cytofix/Cytoperm (BD Biosciences) according to manufacturer's instructions. Later, cells were stained with antimouse IFN-γ-APC, IL-2-PE and TNF-α-PECy7 (Biolegend, 1 μg·mL^-1^) dispersed in Perm Wash Buffer upon incubation for 20 min at 4 °C. Then, cells were washed with Perm Wash Buffer and analyzed using the Aurora spectral cytometer (Cytek). Unstained and single stained cells treated with CpG and COVA were used as controls to unmix the different channels with SpectroFlo V2.2 software. For the gating strategy, live CD3^+^ T-cells were identified and the expression of TNF-α, IL-2 and IFN-γ was analyzed within this population. The data was evaluated using FCS Express 7 (De Novo Software). Results were expressed as % TNF-α+, IL-2 or IFN-γ+ cells within CD3^+^ population.

### 2.13 *In vitro* tumor T cell killing assay (BMDC:CD8+ T cell: B16-OVA coculture)

B16-OVA cells were a kind gift from Prof K. Wucherpfennig's lab at Dana Farber Cancer Institute, and were cultured in DMEM with 4.5 g·L^-1^ of glucose, 2 mM glutamine and 5 mM sodium pyruvate (ThermoFisher) supplemented with 0.4 mg mL^-1^ geneticin (ThermoFisher) and 10% Fetal Bovine Serum (Sigma Aldrich). Cells were used in passages 10–13.

For the *in vitro* tumor killing assay, B16-OVA cells were labeled with CFSE 5 μM as previously indicated. Cells were seeded in tissue culture treated plates (80,000 cells per well in 48-well plates, 200,000 cells per well in 24-well plates and 400,000 cells per well in 12-well plates). Then, BMDC and CD8^+^ T cells (from OT-I or C57BL/6 mice) previously coincubated, as mentioned in section *Ex vivo BMDC:CD8+ T cell coculture*, were added to B16-OVA cells and incubated for 48 or 72 h. Next, cells were harvested using 0.25% v/v trypsin (Sigma Aldrich). Then, cells were resuspended in PBS and stained with LIVE/DEAD™ Fixable Near-IR (ThermoFisher) according to manufacturer's instructions. Later, cells were analyzed using the Aurora spectral cytometer (Cytek). Unstained cells and single-stained controls were used to unmix the different channels with SpectroFlo V2.2 software. For the gating strategy, CFSE + cells were identified as B16-OVA and the cell death was analyzed according to LIVE/DEAD Fixable Near-IR signal within this population. Results were expressed as % cell viability, refering to % of alive cells, within CFSE + population, thus B16-OVA cells.

### 2.14 Functional integrity of MNP-CpG-COVA formulation after intravenous mice injection

MNP-CpG-COVA was administered intravenously (5 mg Fe MNP-CpG-COVA per animal, 100 μL 50 mg Fe·mL^-1^; 125 nmol COVA and 125 nmol CpG) to a 6-week old female C57BL/6 mouse. Thirty minutes after injection, the animal was sacrificed by terminal anesthesia and ∼1 ml of blood was collected *via* cardiac puncture. Plasma, containing MNP-CpG-COVA particles, was isolated by centrifugation of whole blood (2000 g, 10 min) and the concentration of MNP-CpG-COVA nanoparticles was quantified by the Ferrozine Assay. Next, BMDCs were incubated with MNP-CpG-COVA isolated from plasma to assess their ability to induce BMDC maturation and antigen presentation *in vitro*, as described in previous sections.

### 2.15 *In vivo* uptake of MNP-CpG-COVA by spleen cells

To investigate which spleen cells internalized MNP-CpG-COVA, female 6-week-old C57BL/6 mice (n = 15) were divided into three groups (untreated, bolus, and MNP-CpG-COVA, 5 animals per group). The corresponding treatments were injected intravenously (bolus COVA 125 nmol, CpG 125 nmol; MNP-CpG-COVA 5 mg Fe, COVA 125 nmol, CpG 125 nmol). Mice were euthanized 3 days later, and the spleens were isolated and processed. The spleens were disrupted, erythrocyte lysis buffer was used (Red Blood Cell Lysis Buffer, Thermofisher), and the MACS Magnetic Separator (Miltenyi Biotec) was employed to separate the cells that contained MNP-CpG-COVA. Cells were counted in a Neubauer chamber, washed twice with PBS and stained with LIVE/DEAD™ Fixable Blue (ThermoFisher) according to manufacturer's instructions. Then, cells were washed three times with FACS buffer (PBS, 1% BSA) and finally resuspended in 50 μL of FACS buffer containing 5 μg·mL^-1^ antimouse CD16/CD32 monoclonal antibody (ThermoFisher) for 5 min at 4 °C. After, the staining solution containing antibodies for several cell surface markers of spleen cells was added to 50 μL of FACS buffer containing the antibodies at a final concentration of 1 μg·mL^-1^. The panel of labeled antibodies included antimouse CD45-PerCp, CD19-FITC, CD11c-BV421, CD209-PE, CD-169-APC, F4/80-BV650, CD3-PECy5 and MHCII(IA/IE)-BV480 (all obtained from Biolegend). These antibodies were incubated for 30 min at 4 °C. Then, cells were washed three times with FACS buffer and analyzed using the Aurora Spectral Cytometer (Cytek). Unstained and single-stained controls of cells were used to unmix the different channels with SpectroFlo V2.2 software. For the gating strategy, live CD45^+^ cells were identified as immune cells. Then, CD45^+^ CD19^+^ cells were classified as B cells. Among CD45^+^ CD19^−^population, the expression of CD3 was employed to differentiate the lymphocytes. Then, the expression of CD11c, F4/80 and MHCII was analyzed to differentiate between macrophages and dendritic cells. Lastly, CD209 and CD169 expressions were studied to identify different subsets of macrophages. The results were analyzed using FCS Express 7 (De Novo Software).

### 2.16 Study of the immune response and biodistribution of MNP-CpG-COVA *in vivo*

6 weeks old female C57BL/6 mice were divided in five groups (Untreated, 5 mg Fe + 1 boost, 5 mg Fe + 2 boost, 0.5 mg Fe+ 1 boost and 0.5 mg Fe + 2 boost, n = 5 per group). The animals were immunized with MNP-CpG-COVA (day 0) at the mentioned concentrations (5 mg and 0.5 mg Fe MNP-CpG-COVA; respectively) and the boosters were administered after 7 days (1 boost) and 14 days (2 boost). To analyze the immune response generated, blood samples from mice's cheek were recollected (⁓140 μL) on days 7, 14, 21, and 28. Erythrocyte lysis buffer (Red Blood Cell Lysis Buffer, Thermofisher) was used to eliminate red blood cells according to manufacturer's instructions. To evaluate the immune response in the peripheral blood mononuclear cells (PBMCs), two different strategies were performed:

- *Tetramer analysis*: blood cells were stained with APC conjugated SIINFEKL-MHC tetramer (TetramerShop) according to manufacturer's instructions. Briefly, blood cells were washed twice with PBS containing 2% FBS and resuspended in 50 μL PBS. Then, 5 μL of the tetramer were added and incubated for 15 min at 37 °C in the dark. Next, cells were washed twice with PBS and stained with Live/Dead Blue™ fixable blue (Thermofisher) according to manufacturer's instructions. Later, samples were washed three times with FACs buffer (PBS 1% BSA). Then, cells were resuspended in 50 μL of FACS buffer containing 5 μg·mL^-1^ antimouse CD16/CD32 monoclonal antibody (ThermoFisher) for 5 min at 4 °C. Next, 50 μL of FACS buffer containing antimouse CD3-PE-Cy5, CD8a-BV605 and CD4-BV711 (1 μg·mL^-1^ each, Biolegend) were added, and the samples were incubated for 30 min at 4 °C in the dark. Later, cells were washed three times with FACS buffer, and fixed with Cytofix/Cytoperm (BD Biosciences) following manufacturer's instructions. Lastly, the samples were analyzed using the Aurora spectral cytometer (Cytek). Unstained cells and single stained controls of cells were used to unmix the different channels with SpectroFlo V2.2 software. For the gating strategy, live CD3^+^ CD8^+^ cells were selected to evaluate the expression of Tetramer + population, as indicated in Fig. S15. The results are expressed as % Tetramer+ within CD3^+^ CD8^+^ cells. Data were analyzed using FCS Express 7 (De Novo Software).
- *Peptide restimulation of circulating PBMC*: blood cells were pulsed with 10 μg·ml^-1^ of COVA for 1.5 h at 37 °C in T cell culture media. Subsequently, GolgiPlug (BD Biosciences; 1 μL per well) was added to stop cytokine secretion and incubated for 4 h at 37 °C, 5% CO_2_. Next, cells were washed three times with PBS and stained with Live/Dead Blue™ fixable blue (Thermofisher) according to manufacturer's instructions. Later, cells were washed three times with FACS buffer (PBS 1% BSA). Then, samples were resuspended in 50 μL of FACS buffer containing 5 μg·mL^-1^ antimouse CD16/CD32 monoclonal antibody (ThermoFisher) for 5 min at 4 °C. Next, 50 μL of FACS buffer containing antimouse CD3-PE-Cy5, CD8a-BV605, CD4-BV711, CD44-BV785 and CD62L-FITC (1 μg·mL^-1^ each, Biolegend) were added, and the samples were incubated for 30 min at 4 °C in the dark. Later, cells were washed three times with FACS buffer, and fixed with Cytofix/Cytoperm (BD Biosciences) following manufacturer's instructions. Then, cells were stained with antimouse IFN-γ-PE, GzmB APC Fire 750, TNF-α-PE-Cy7 and IL-2-AF700 (Biolegend, 1 μg·mL^-1^) dispersed in Perm Wash Buffer upon incubation for 20 min at 4 °C. Lastly, the samples were analyzed using the Aurora spectral cytometer (Cytek). Unstained cells and single stained controls of cells were used to unmix the different channels with SpectroFlo V2.2 software. For the gating strategy, indicated in Fig. S15, live CD3^+^ CD8^+^ cells were selected to evaluate the expression of IFN-γ+, GzmB+, TNF-α+, and IL-2+ populations. The results expressed as % cytokine+ within CD3^+^ CD8^+^ cells. Additionally, CD44 and CD62L markers were also analyzed in live CD3^+^ CD8^+^ cells and classified as näive (T_N_, CD44^−^ CD62L+), central memory (T_CM_, CD44^+^ CD62L+), and effector memory (T_EM_, CD44^+^ CD62L-). The results expressed as % T cells within CD3^+^ CD8^+^ cells. Data were analyzed using FCS Express 7 (De Novo Software).

On day 35, mice were euthanized (CO_2_ inhalation). Lungs, kidneys, livers, and spleens were recollected and lyophilized. Then, organs were digested in aqua regia (HCl: HNO_3_ 3:1) for a week. The iron content was measured by Inductively coupled plasma optical emission spectrometry (ICP-OES). The results were expressed as pg Fe per g dried organ.

### 2.17 Therapeutic tumor studies

7 weeks-old female C57BL/6 mice were injected with a single-cell suspension of 1 × 10^5^ B16-OVA cells in 100 μL cold PBS subcutaneously in the back on day 0. On day 5, animals were randomly distributed to experimental groups. The treatments were administered in a double-blind fashion, wherein the individuals responsible for injections and tumor measurements were unaware of the specific treatments being administered. Tumor volume was monitored using a calliper and calculated as (length) x (wide) x (height) x π/6. Mice were euthanized when the tumors were necrotic or reached 17 mm in any three dimensions and according to humane endpoint described by IACUC standards. The formulations tested include two doses of MNP-CpG-COVA (high dose: 5 mg Fe, 125 nmol CpG, 125 nmol COVA; low dose: 0.5 mg Fe, 12.5 nmol CpG, 12.5 nmol COVA) and a negative control of MNP functionalized with CpG and an irrelevant peptide sequence which should not induce any antitumoral response against B16-OVA cells (MNP-CpG-VSV; Vesicular Stomatitis Virus (VSV) peptide. sequence include in Table S1).

## 3 Results

### 3.1 Synthesis, functionalization and characterization of MNP and *in vitro* biocompatibility studies with RBC and BMDC

Magnetic nanoparticles (MNP) composed of 14 nm γ-Fe_2_O_3_ cores coated with carboxymethyldextran were prepared (Fig. S1A). MNP were stable colloidal formulations in water with a monodispersed hydrodynamic size of ⁓ 130 nm (Fig. S1B) and negative surface charge (Fig. S1C). No endotoxins were detected in the formulation (<0.25 EU·mL^-1^) (Fig. S1D).

*In vitro* biocompatibility studies with red blood cells (RBC) suggested that MNP were only hemolytic (% hemolysis >5% after 3 h of incubation [49]) at concentrations higher than 0.25 mg Fe·mL^-1^ (Fig. S2A). The % of hemolysis was always below 10% at all incubation times (1, 3 and 24 h) and concentrations (0.01–2 mg Fe·mL^-1^) tested (Fig. S2A). Cytocompatibility studies in BMDC indicated that MNP were not toxic in a wide range of concentrations (0.01–0.25 mg Fe·mL^-1^; incubation times 4, 24, and 48 h) (Figs. S2B–C). Next, cell-cycle studies confirmed that MNP did not significantly affect the timing and frequency of DNA duplication and cell division at short incubation times (4 and 24 h) and only at high concentrations (0.1 and 0.25 mg Fe·mL^-1^) and after 48 h of incubation there was a notable reduction of cells entering G2/M phase (Fig. S2D). Internalization studies in BMDC evidenced that uptake was time and concentration-dependent (Figs. S3A–C) and the maximum load of nanoparticles was achieved after 40 h of incubation (Fig. S3B).

**Fig. 1:**
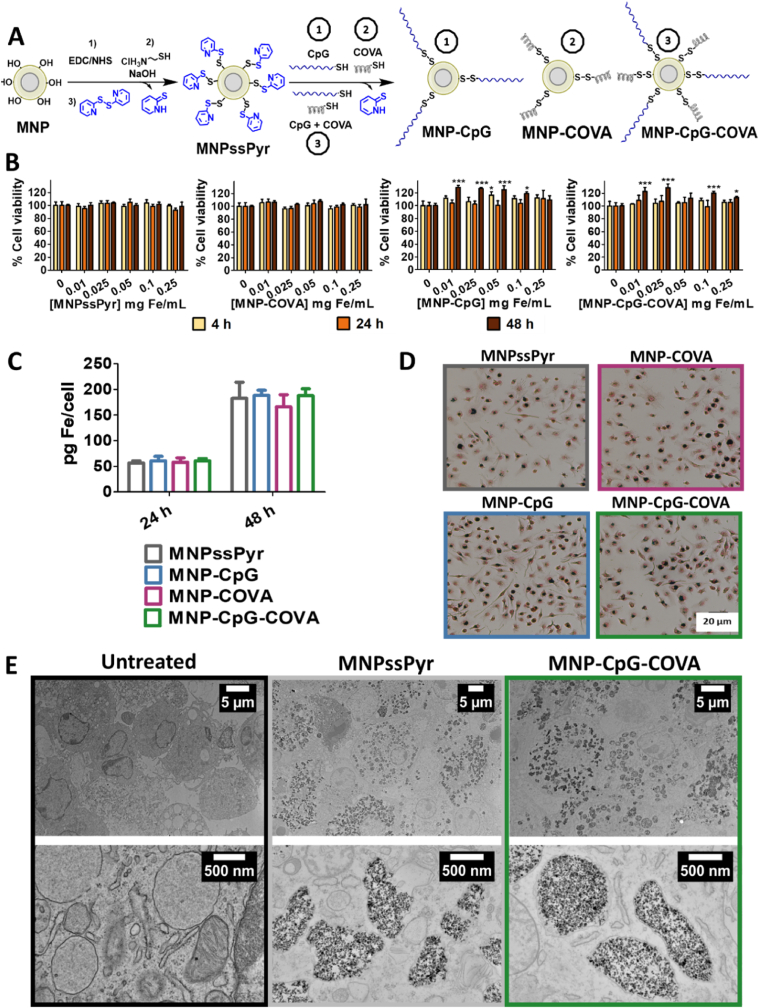
Functionalized MNP. (A) Scheme of MNP functionalization with CpG and COVA. (B) Cytotoxicity evaluation of functionalized nanoparticles in BMDC by Alamar Blue after 4, 24 and 48 h of incubation. Data represent mean ± SD (n = 6). For statistical analysis, one way ANOVA was performed (untreated *vs* each concentration of MNP). *p < 0.05, **p < 0.01, ***p < 0.001. (C) Quantification of iron in BMDC incubated with functionalized MNP (0.05 mg Fe·mL^-1^) for 24 h and 48 h by ferrozine assay. After 24 h and 48 h of incubation of BMDC with functionalized MNP (0.05 mg Fe·mL^-1^). Data represent mean ± SD (n = 3). (D) Prussian Blue staining photos of BMDC incubated with functionalized MNP (0.05 mg Fe·mL^-1^) for 48 h. (E) TEM images of BMDC untreated and incubated with MNPssPyr or MNP-CpG-COVA (0.05 mg Fe·mL^-1^) for 48 h.

Once we attested the biocompatibility of MNP, we proceeded with their functionalization to conjugate the adjuvant oligonucleotide CpG and the model antigen peptide COVA *via* disulfide bonds, as described in Fig. 1A. Our proposed functionalization procedure enables us to monitor the process by quantifying the release of pyridinthione into the medium. This allows for the precise measurement of the amount of CpG (2.5 μmol per g Fe) and COVA (2.5 μmol per g Fe) attached to the system. In all cases, MNP functionalization was associated with changes in hydrodynamic size and ζ potential (Table S2), but it did not alter their cytocompatibility (Fig. 1B) and internalization in BMDC (Fig. 1C and D). We confirmed the intracellular localization of MNP inside cytoplasmic vesicles (Fig. 1E).

### 3.2 *In vitro* studies of BMDC maturation and antigen presentation upon incubation with MNP formulations

**Fig. 2:**
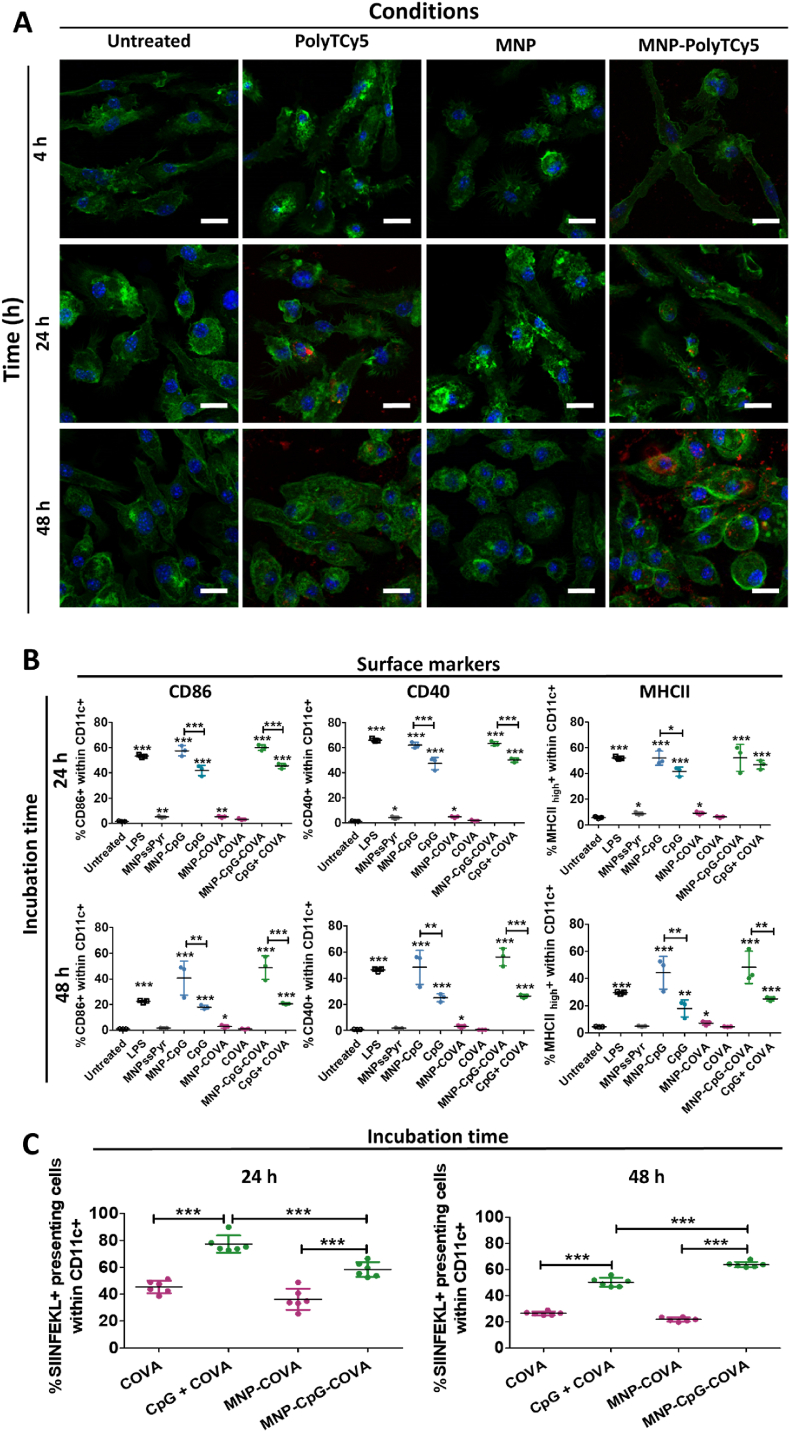
(A) Confocal images of BMDC untreated and incubated with polyTCy5, MNP or MNP-PolyTCy5 after 4, 24 and 48 h of incubation (0.05 mg Fe·mL^-1^, 0.125 μM PolyTCy5). Phalloidin: green; DAPI: blue; Cy5: red. Scale bar: 10 μm. (B–C) *In vitro* studies of the effects of functionalized MNP in BMDC. Conditions tested: [LPS] 5 ng·mL^-1^, [MNP] 0.05 mg Fe·mL^-1^, [CpG] 0.125 μM, [COVA] 0.125 μM. (B). Evaluation of maturation surface markers CD86, CD40 and MHCII expression in CD11c + BMDC after 24 and 48 h of incubation with the corresponding treatments. Data represent mean ± SD (n = 3). For statistical analysis, one-way ANOVA was performed (untreated *vs* each treatment). *p < 0.05, **p < 0.01, ***p < 0.001. (C) Percentage of CD11c + BMDC that present OVA_257-264_ (SIINFEKL) bound to MHCI after 24 and 48 h of incubation with the corresponding treatments. Data represent mean ± SD (n = 6). Statistical analysis was performed with one-way ANOVA test. ***p < 0.001.

To assess if functionalized MNP could deliver their cargo to BMDC, we conjugated the fluorescently-tagged oligonucleotide PolyTCy5 (MNP-PolyTCy5) to their surface following the same chemistry as above. First, *in vitro* release studies confirmed that the cargo was released when the disulfide bonds were broken by the addition of dithiothreitol (Fig. S4). Then, confocal imaging of BMDC incubated with MNP-PolyTCy5 indicated that the internalization of the oligonucleotide was higher when linked to MNP than in the free form, especially after 48 h of incubation (Fig. 2A). Thus, we expected the highest effect of the modified MNP to occur at that time point.

Next, we tested the levels of activation in BMDC triggered by MNP-CpG, MNP-COVA, and MNP-CpG-COVA (0.05 mg Fe·mL^-1^, 0.125 μM CpG, 0.125 μM COVA) after 24 and 48 h of incubation (Fig. 2B). As expected, CpG alone or in combination with COVA was able to trigger the upregulation of BMDC maturation surface markers. However, formulations in which CpG was conjugated to the nanoparticles (MNP-CpG and MNP-CpG-COVA) induced significantly higher levels of maturation markers that were also more sustained over time (see data at 48 h incubation) than the soluble forms.

The self-adjuvant properties of the nanoparticles were also assessed (Fig. S5A). The results confirmed that bare MNP were also able to trigger the expression of maturation markers in a dose-dependent manner (Fig. S5A). However, MNP that went through the washing steps needed for the functionalization process (MNP washed) and those further functionalized with 2-pyridinethione groups (MNPssPyr) induced much lower activation of co-stimulatory markers. This reduced immune activation was likely associated with a reduction in the organic layer that takes place during their processing (Fig. S5B), since we did not observe significant changes in particle internalization among different formulations (Fig. S5C). Overall, this data suggests that BMDC activation triggered by MNP-CpG and MNP-CpG-COVA is likely primarily driven by the adjuvant cargo.

We next studied the presentation of the peptide antigen OVA_257-264_ (SIINFEKL) through MHC I in BMDC after 24 and 48 h of incubation (Fig. 2C). The combination of COVA and CpG resulted in higher presentation levels than the peptide alone at both time points tested, either in their soluble form or coupled to MNP. Of note, the percentage of SIINFEKL + BMDC was sustained for at least 48 h at ∼60% when cells were treated with MNP-CpG-COVA, whereas it decreased sharply from 24 to 48 h in the rest of the conditions (Fig. 2B).

### 3.3 *In vitro* CD8^+^ T cell expansion and activation studies

**Fig. 3:**
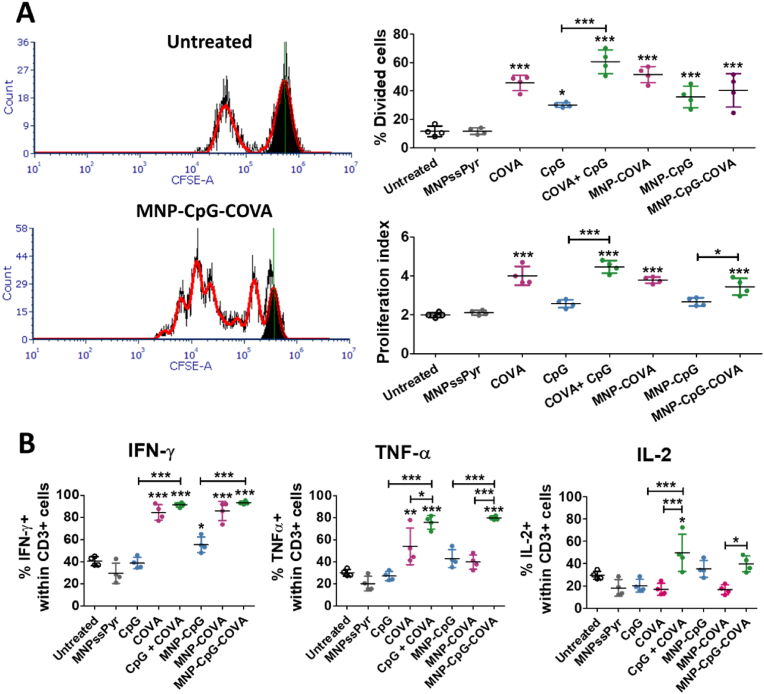
*In vitro* studies of CD8^+^ OT-I T cells response to BMDC coculture for 48 h (ratio T cells: BMDC 1:1). BMDC were previously incubated with the indicated treatments for 6 h. Conditions tested: [LPS] 5 ng·mL^-1^, [MNP] 0.05 mg Fe·mL^-1^, [CpG] 0.125 μM, [COVA] 0.125 μM. (A) T cell proliferation assay. Representative flow cytometry histogram showing CFSE fluorescence of CD8^+^ OT-I T cells (left) and calculated percentage of divided cells and proliferation index (right). Data represent mean ± SD (n = 4). For statistical analysis, One way ANOVA was performed (untreated *vs* each treatment). *p < 0.05, ***p < 0.001. (B) Percentage of IFN-γ, TNF-α and IL-2 positive cells in CD8^+^ OT-I T cells after intracellular cytokine staining. Data represent mean ± SD (n = 4). Statistical analysis was performed with One Way ANOVA test (untreated *vs* each treatment). *p < 0.05, **p < 0.001, ***p < 0.001.

To assess if our proposed formulation was able to trigger a cytotoxic T cell response, we evaluated the ability of treated BMDC to induce T cell proliferation and activation *in vitro*. First, the division of OT-I CD8^+^ T cells, which express the transgenic OVA_257-264_ specific TCR, was analyzed after two days of coculture with BMDC pulsed with the formulations (Fig. 3A). The results suggested that BMDC treated with the adjuvant CpG, the antigen peptide COVA or both, in their soluble form or anchored to MNP, induced the division of OT-I CD8^+^ T cells (higher % of divided cells). In terms of proliferation index, expressed as the total number of cell divisions divided by the number of cells that went into division, the treatment with COVA alone or in combination with CpG promoted higher values. Additionally, the production of IFN-γ, TNF-α and IL-2 by CD8^+^ OT-I T cells was tested to estimate their levels of activation (Fig. 3B). The production of these cytokines by T cells was enhanced when BMDC were treated with the soluble CpG and COVA in combination, or MNP-CpG-COVA. Thus, our system induced expansion and activation of antigen-specific CD8^+^ T cells, which is suggestive of significant cytotoxic activity.

### 3.4 *In vitro* tumor killing assay

**Fig. 4:**
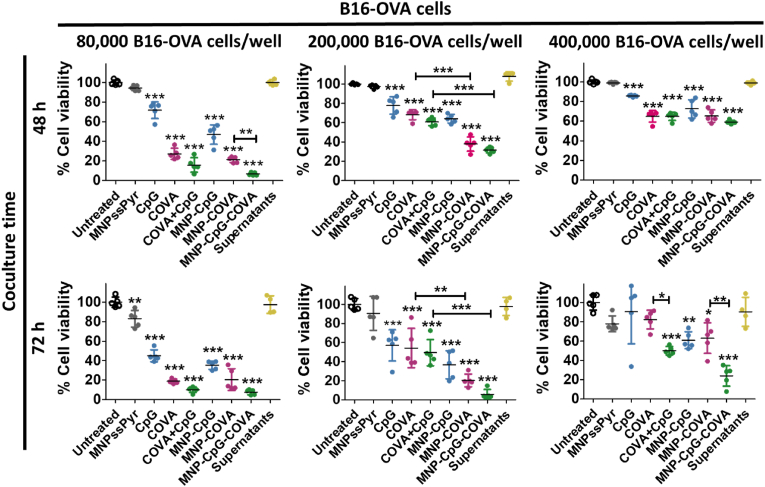
*In vitro* tumor CD8^+^ OT-I T cell killing assay. Detection of live and dead B16-OVA cells previously stained with CFSE. Data is represented as percentage of live cells compared to the untreated control (mean ± SD, n = 4). BMDC were treated for 6 h with the corresponding treatments (0.05 mg Fe·mL^-1^; [CpG] 0.125 μM, [COVA] 0.125 μM), coincubated for 48 h with CD8^+^ OT-I T cells and then cocultured for 48 or 72 h with different number of B16-OVA cells (initial BMDC: B16-OVA ratios 1:2, 1:5 and 1:10). Statistical analysis was performed with One Way ANOVA test. *p < 0.05, **p < 0.001, ***p < 0.001.

To investigate the antigen-specific cytotoxic potential of the CD8^+^ OT-I T cells against ovalbumin-expressing melanoma cells (B16-OVA), we cocultured them with different numbers of cancer cells (80,000, 200,000, and 400,000 cells per well) for 48 h and 72 h *in vitro* (Fig. 4). The results revealed that when BMDC were treated with CpG, COVA, or their combination, in their soluble form or coupled to MNP, CD8^+^ OT-I T cells activation resulted in a reduction of B16-OVA cell viability that was dependent on the number of cancer cells. Particularly, the higher the number of B16-OVA cells, the lower the cytotoxic effect of CD8^+^ OT-I T cells. However, the cytotoxic T cell response promoted by MNP-CpG-COVA after 72 h of coculture was consistently very high (∼20% cell viability) regardless of the concentration of B16-OVA cells tested. Furthermore, to confirm that this was an effect produced by BMDC uptake of intact MNP-CpG-COVA and not of potentially realeased CpG and COVA to the culture media, MNP-CpG-COVA were incubated for 48 h in cell culture media, centrifuged to remove the particles, and BMDC were treated with the resulting supernatant. The data revealed that the supernatant of MNP-CpG-COVA did not lead to B16-OVA cell death at any ratio tested, hence ruling out a potential premature release of the cargo from MNP.

We confirmed this tumor killing activity to be antigen specific, repeating the above studies with CD8^+^ T cells from C57BL/6 mice that do not express the transgenic OVA_257-264_ specific TCR (Fig. S6). In this case, the C57BL/6 T cells did not induce a cytotoxic response against B16-OVA cells.

### 3.5 Robustness of disulfide bonds in the bloodstream

To assess the robustness of the disulfide bonds in the blood circulation, MNP-CpG-COVA were intravenously injected in mice (100 μL 50 mg Fe·mL^-1^; 5 mg Fe) and isolated from blood plasma 30 min after the injection. Next, we tested the isolated particles in *in vitro* BMDC exposure experiments, at the same concentrations as in previous studies, and measured the levels of activation markers and antigen presentation after 24 and 48 h of incubation (Fig. S7). The levels of CD86, CD40 and MHCII, as well as the levels of SIINFEKL presentation, were similar to those obtained with freshly prepared MNP-CPG-COVA (Fig. 2), suggesting that the integrity of the disulfide bonds was maintained for at least 30 min in the bloodstream.

### 3.6 *In vivo* biodistribution studies and the immune response evaluation of MNP-CpG-COVA

**Fig. 5:**
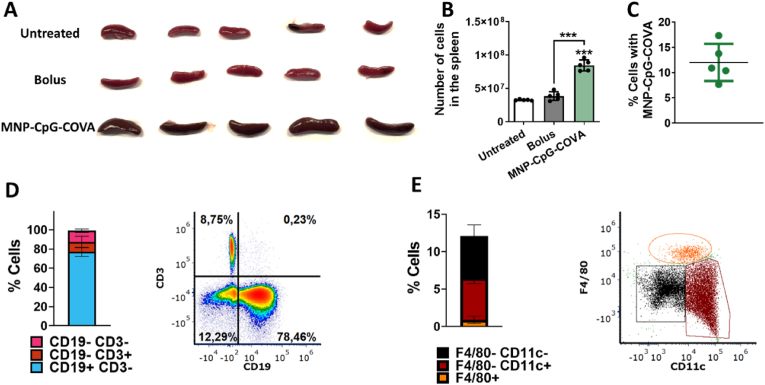
Biodistribution of MNP-CpG-COVA in the spleen. (A) Image of isolated spleens from mice untreated, treated with a bolus vaccine ([CpG] 0.125 μM, [COVA] 0.125 μM), and treated with MNP-CpG-COVA (5 mg Fe, ([CpG] 0.125 μM, [COVA] 0.125 μM), three days after the injection. (B) Number of cells in the spleen (mean ± SD, n = 5 per group). Statistical analysis was performed with One Way ANOVA test. ***p < 0.001. (C) Percentage of spleen cells that contained MNP-CpG-COVA. (D) Percentage of spleen cells with MNP-CpG-COVA as a function of CD19 and CD3 markers expression (left) and representative quadrants of cells populations (right). (E) Percentage of spleen cells with MNP-CpG-COVA as a function of F4/80 and CD11c expression within CD19^−^ CD3^−^population (left) and representative quadrants of cells populations (right).

Firstly, the biodistribution of MNP-CpG-COVA in the spleen was analyzed since this organ is populated by immune cells involved in the antitumor response [50]. MNP-CpG-COVA (5 mg Fe, 125 nmol CpG, 125 nmol COVA; 100 μL 50 mg Fe·ml^-1^) were administered intravenously (n = 5) and the spleen was isolated three days after (untreated and bolus injected mice were used as controls). The spleen's size of mice inoculated with the MNP-CpG-COVA was significantly larger (Fig. 5A) and showed increased cellularity (Fig. 5B) than the untreated and bolus controls (125 nmol CpG, 125 nmol COVA). Then, the spleen cells containing MNP-CpG-COVA were magnetically separated from the rest and counted. The results suggested that the formulation was internalized by 12 ± 3.68% of the spleen cells (Fig. 5C). Next, staining of several cell surface markers suggested that MNP-CpG-COVA were preferentially taken by B cells (CD19^+^ CD3^−^, 76.91 ± 4.67%), and only a small fraction was in contact with T cells (CD19^−^ CD3^+^, 11.73 ± 1.78%) and CD19^−^ CD3^−^cells (10.46 ± 5.86%) (Fig. 5D). The staining was validated by confirming the proportion of these cells' population in the rest of the conditions tested (Fig. S8A). The fraction of CD19^−^ CD3^−^was expected to be constituted by dendritic cells and macrophages, among other immune cells. The expression of CD11c and F4/80, two classical markers of dendritic cells and macrophages, respectively [51,52], were then studied. Red pulp macrophages (F4/80+ that were also MHCII+, Fig. S8C) constituted a small fraction (0.90 ± 0.49%) (Fig. 5E). However, this population was absent after magnetic separation of spleen cells with MNP-CpG-COVA (Fig. S8B). It is possible that the magnetic cell isolate was contaminated with red pulp macrophages due to their superparamagnetic behavior related to iron accumulation [53]. Thus, the percentage of these cells that incorporated MNP-CpG-COVA cannot be determined by this technique. Regarding F4/80- CD11c + cells, they constituted half of CD19^−^ CD3^−^cell fraction (5.60 ± 0.76%) (Fig. 5E), but only approximately half of them were MHCII+ (2.43 ± 0.35%) (Fig. S8D), thus conventional dendritic cells. Additionally, in the fraction of F4/80- CD11c-cells, MNP-CpG-COVA was internalized by a small fraction of marginal zone macrophages (1.44 ± 0.24%) and a negligible amount of metallophillic macrophages (0.10 ± 0.1%) (Fig. S8E).

**Fig. 6:**
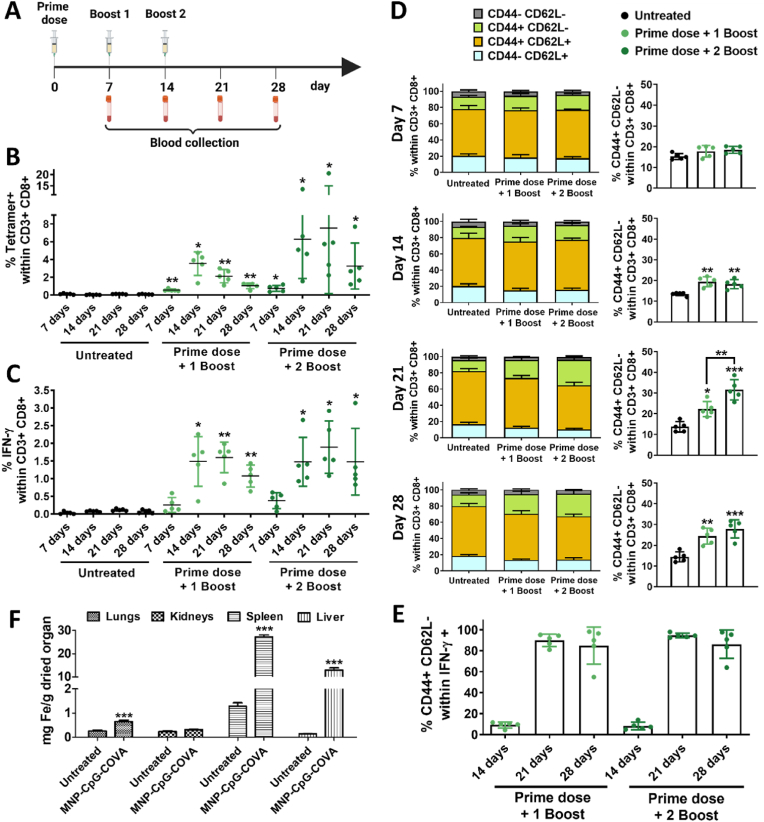
*In vivo* evaluation of MNP-CpG-COVA (5 mg Fe, 125 nmol CpG, 125 nmol COVA) effects in the immune response and study of its biodistribution in C57BL/6 mice. (A) Schematic timeline representation of mice vaccination and blood extraction for immune response analysis. (B) Percentage of tetramer + CD8^+^ T cells. (C) Percentage of IFN-γ+ CD8^+^ T cells. (D) Division of CD8^+^ T cells according to CD44 and CD62L expression (left) and percentage of CD8^+^ effector T cells (CD44^+^ CD62L-) (right). (E) Percentage of effector T cells (CD4+, CD62L-) within IFN-γ+ CD8^+^ T cells. (F) Representation of MNP-CpG-COVA biodistribution in the lungs, kidneys, spleen and liver as a function of the iron content in the organs. Results are expressed as mg iron per g dried organ. Statistical analysis was performed with One Way ANOVA test. *p < 0.05, **p < 0.001, ***p < 0.001.

The ability of MNP-CpG-COVA to induce a cytotoxic T lymphocyte (CTL) response against the model antigen OVA was next studied. Two different doses (5 mg Fe, 125 nmol COVA, 125 nmol CpG; and 0.5 mg Fe, 12.5 nmol COVA, 12.5 nmol CpG) and vaccination strategies (prime dose and one booster 7 days later; prime dose and two boosters 7 and 14 days later) were compared. Circulating peripheral blood mononuclear cells (PBMCs) were analyzed 7, 14, 21, and 28 days after the first immunization with MNP-CpG-COVA, as described in Fig. 6A.

Mice immunized with the higher dose of MNP-CpG-COVA (5 mg Fe) generated a notable percentage of tetramer+ (Fig. 6B) and IFN-γ+ CTLs (Fig. 6C) that significantly improved 14 days after the first immunization, thus after boosters’ administration. The administration of a second booster contributed to maintaining high levels of tetramer + CTLs levels over time (21 and 28 days), whereas similar percentages of IFN-γ+ CTLs were detected with one and two boosters at all time points tested. The percentage of Granzyme B+ cells slightly increased 7 days after the first immunization (0.32 ± 0.23% and 0.45 ± 0.30%, Fig. S10B) and TNF-α after 28 days (0.34 ± 0.22% with one booster, 0.71 ± 0.62% with two boosters, Fig. S10A) and the IL-2+ CTLs (Fig. S10C) did not markedly expand under the conditions tested. The comparative analysis of CD8^+^ T cells subsets in PBMCs (Fig. 6D) showed that the effector subset (CD44^+^ CD62L-) constituted ⁓ 20% of CD8^+^ T cells, with levels being maintained over time after the administration of the first booster. The second booster substantially increased the percentage of effector T cells on day 21 after the first immunization (31.56 ± 4.90% *vs* 22.28 ± 3.68%). Notably, the IFN-γ CD8^+^ T-cells were mainly effector T cells on days 21 and 28 after the first inoculation (Fig. 6E), suggesting a long-lasting effect of the immunization on CTLs function.

The immunization with a lower dose of MNP-CpG-COVA (0.5 mg Fe) resulted in lower levels of tetramer+ (Fig. S9A) and IFN-γ (Fig. S9B) CTLs. The percentage of TNF-α+ CTLs (Fig. S10D) slightly increased after the administration of three doses and after 28 days of the first immunization (0.34 ± 0.23%), whereas no changes in the levels of GzmB+ (Fig. S10E) and IL-2+ (Fig. S10F) CTLs were observed. The analysis of CD8^+^ T cells subsets in PBMCs (Fig. S9C) indicated a moderate increase of effector T cells population 7 days after the first immunization (20.53 ± 1.28 and 20.23 ± 1.30%) that was maintained over time with the administration of one or two boosters, but which returned to untreated levels after 28 days.

Overall, the results suggested a dose-response effect of MNP-CpG-COVA, which was especially noteworthy with the higher dose (5 mg Fe). Importantly, the treatments were not associated with mice weight loss (Fig. S11), indicating the absence of toxicity.

Next, we investigated the biodistribution of MNP-CpG-COVA. These studies attested that the nanoparticles were preferentially accumulated in the spleen and liver, with a negligible amount in the lungs and no accumulation in the kidneys (Fig. 6F).

To assess the antigen-specific anti-tumoral effect of MNP-CpG-COVA, we initially inoculated mice with B16-OVA cells and subsequently treated them with either MNP-CpG-COVA or MNP-CpG-VSV, the latter being a formulation containing an antigen from the vesicular stomatitis virus and serving as a non-antigen specific control (Fig. S12A). The results suggest that MNP-CpG-COVA formulation retards tumor growth and enhances mouse survival in a dose-dependent manner (Figs. S12B and S12C), aligning with the results obtained in the immunization studies (Fig. 6A-E, S9, S10). The median survival of untreated (29 days) and MNP-CpG-VSV treated mice (25 days) were lower than both concentrations tested of MNP-CpG-COVA treated animals (low dose 32.5 days; high dose 37 days). Remarkably, one-third of the animals immunized with the high dose of MNP-CpG-COVA remained alive 45 days after the inoculation (Fig. S12B).

## 4 Discussion

This study demonstrates the potential of functionalizing MNP *via* disulfide bonds for the generation of an antigen-specific cancer vaccine. Nanoparticles (e.g., liposomes, polymeric nanoparticles, gold nanoparticles) have been widely explored for the delivery of peptide-based vaccines mainly with the aim of protecting antigens from degradation but also to control their release [54]. The electrostatic and covalent functionalization of magnetic nanoparticles with model antigens such as OVA protein [31,32,55], OVA_257-264_ peptide [56,57], and with adjuvants such as CpG [33], or with the combination of both in the same nanoparticle [58], have been explored in the literature. However, to the best of our knowledge, there are no previous studies evaluating the use of a disulfide bond as a stimuli-responsive linker for MNP-based cancer vaccine functionalization. In this study, we demonstrated that MNP presented good colloidal stability (Fig. S1) and biocompatibility *in vitro* (Fig. 1; and Figs. S2 and S3). Their functionalization resulted in BMDC activation (*in vitro*) even after 48 h of incubation, when the response triggered by the soluble components had already decayed (Fig. 2), which could be related to the gradual release of the adjuvant and antigen from the nanoparticles and their continuous internalization. Moreover, MNP-CpG-COVA conserved their capacity to activate BMDC even after 30 min in the blood circulation, as tested *in vitro* (Fig. S7). By functionalizing the nanoparticles *via* disulfide bonds, the premature release of CpG and COVA is avoided. The proliferation and initiation of antigen-specific T cell proliferation and the initiation of their effector function (Fig. 3) was validated with the potent antigen-specific antitumor response observed *in vitro* (Fig. 4, and Fig. S6). Overall, the *in vitro* studies suggest that functionalized MNP promote a more potent and sustained antitumor response in comparison with their soluble counterparts. The intravenous administration of MNP-CpG-COVA can lead to interactions with diverse immune cells within the bloodstream, including peripheral macrophages. These immune cells have the capacity to migrate from the bloodstream to secondary lymphoid organs, such as the spleen, where they play a crucial role in promoting the activation of adaptive immune responses [3]. In fact, the administration of MNP-CpG-COVA resulted in an increase in the number of cells in the spleen and a substantial enlargement of the organ in comparison with the untreated mice and the bolus inoculation (Fig. 5A), which is usually correlated with an active immune response [59,60]. Interestingly, MNP-CpG-COVA were preferentially found in B cells, and, to a lesser extent, in T cells, dendritic cells, and macrophages (Fig. 5D–E, and Fig. S8). It is known that B cells are phagocytic, capable of presenting antigens, releasing cytokines, and directly lysing tumor cells [[61], [62], [63]], so future studies looking at the role of B cells in the uptake and processing of MNP-CpG-COVA could be interesting to determine the contribution of B cell immunity to the immune response. MNP associated with T cells are most probably adsorbed on their surface, as per previous reports [64]. Moreover, MNP were also internalized by macrophages and dendritic cells, being both able to trigger a T cell response by acting as APCs [65,66]. Of note, only a small fraction was found in marginal zone macrophages (MZM) despite the fact that it has been reported that negatively charged nanoparticles, like MNP-CpG-COVA, target specific scavenger receptors present in MZM, such as MARCO [67,68]. Additional biodistribution studies confirmed that MNP-CpG-COVA were preferentially located in the spleen and liver (Fig. 6F), two organs populated by immune cells [50,69,70]. Considering these results, it is expected that the systemic anti-tumor immune response induced by MNP-CpG-COVA involves a complex interplay between different immune cell populations, which warrants further investigation.

The evaluation of the T cell mediated immunity *in vivo* (Fig. 6, S9, S10) confirmed that the production of antigen-specific (tetramer+) and active (IFN-γ+) CTLs was dose-dependent (5 mg Fe > 0.5 mg Fe), which correlates with the deceleration of tumor growth and extended survival time (Fig. S12). Importantly, boosters contributed to maintaining the immune response over time, including a higher percentage of effector T cells. Of note, the CTLs activation levels were similar to the ones reported for efficient biomaterials-based cancer vaccines [71,72] and other nanoparticle-based delivery systems [[73], [74], [75]]. However, these materials, in general, require multistep and assiduous synthesis processes that are usually difficult to scale-up [76]. MNP-CpG-COVA can be produced in cost-effective and easy functionalization process, which could be easily adapted for personalized treatments.

Altogether, we have confirmed that MNP can be used to co-deliver antigens and adjuvants to immune cells and promote an efficient anti-tumor response. This work is a stepping stone for the further development of MNP as cancer vaccines. The incorporation of other components onto the nanoparticles *via* the same robust disulfide bonds (e.g., targeting agents such as antibodies or aptamers, multiple adjuvants, and assorted antigens) and/or using the intrinsic magnetic properties of MNP to promote their retention in the target area, to follow their biodistribution by MRI, or to enhance the immune response in combination with mild-magnetic hyperthermia can be explored [17,77].

## 5 Conclusions

This work describes a general and simple strategy to activate anti-tumor immunity by magnetic nanoparticles modified *via* disulfide bonds with an antigen and adjuvant (MNP-CpG-COVA). This approach proved to drive the activation of dendritic cells and, subsequently, T cells to efficiently kill cancer cells *in vitro* in an antigen-specific manner. Moreover, the *in vivo* studies demonstrated that the integrity of the disulfide bonds in the vaccine was maintained in the bloodstream, and the immunization resulted in remarkable levels of active antigen-specific effector T cells, providing a potent therapeutic option. Overall, this work illustrates the high value of magnetic nanoparticles as a therapeutic multifunctionalized platform to enable robust personalized anti-tumor vaccination.

## Credit author statement

Nuria Lafuente-Gómez: conceptualization, data curation, formal analysis, investigation, methodology, visualization, funding acquisition, writing-original draft. Irene de Lázaro: conceptualization, investigation, methodology, supervision, writing-review and editing. Mónica Dhanjani, David García-Soriano, Miguel Sobral and Gorka Salas: investigation, resources, writing-review and editing. David J. Mooney and Álvaro Somoza: conceptualization, methodology, writing-review and editing, funding acquisition, supervision and project administration.

## Declaration of competing interest

The authors declare that they have no known competing financial interests or personal relationships that could have appeared to influence the work reported in this paper.

## Data Availability

Data will be made available on request.
