# Supplementary material for: Multifunctional magnetic nanoparticles elicit anti-tumor immunity in a mouse melanoma model
Source: Mater Today Bio. 2023 Sep 24;23:100817. doi: 10.1016/j.mtbio.2023.100817 (PMC10562177; doi:10.1016/j.mtbio.2023.100817)
Supplement: Multimedia component 1 [file mmc1.docx]

**Supplementary Tables**

|  | **Sequence** |
| --- | --- |
| **CpG** | 5’ Thiol-C6-TTTTT*C*C*A*T*G*A*C*G*T*T*C*C*T*G*A*C*G*T*T 3’ |
| **PolyTCy5** | 5’ Thiol-C6-TTTTTTTTTTTTTTTTTTTTTTTTT-Cy5 3’ |
| **COVA** | CSIINFEKL (H–Cys–Ser – Ile – Ile –Asn– Phe – Glu – Lys – Leu – OH) |
| **VSV** | CRGYVYQGL (H– Cys– Arg– Gly– Tyr–Val– Tyr–Gln–Gly–Leu– OH) |

**Table S1**.- Sequences of the oligonucleotides (CpG and PolyTCy5) and peptide (COVA, VSV) used in the functionalization of MNP. The “*” corresponds to phosphorothioate bond modifications in the oligonucleotide sequence of CpG.

|  | **Hydrodynamic size** | | **ζ potential (mV)** |
| --- | --- | --- | --- |
|  | **Diameter (nm)** | **PDI** |  |
| **MNPssPyr** | 139.80 ± 2.91 | 0.26 ± 0.01 | -24.80 ± 1.80 |
| **MNP-CpG** | 153.10 ± 3.16 | 0.28 ± 0.03 | -29.20 ± 0.90 |
| **MNP-COVA** | 89.79 ± 0.72 | 0.13 ± 0.01 | -28.90 ± 2.07 |
| **MNP-CpG-COVA** | 185.70 ± 10.91 | 0.21 ± 0.05 | -30.10 ± 4.38 |

**Table S2**.- Hydrodynamic size and ζ potential of functionalized magnetic nanoparticles in water (pH 7), 0.05 mg Fe·mL^-1^, 25 ºC. Data represent mean ± SD (n=3).

**Supplementary Figures**


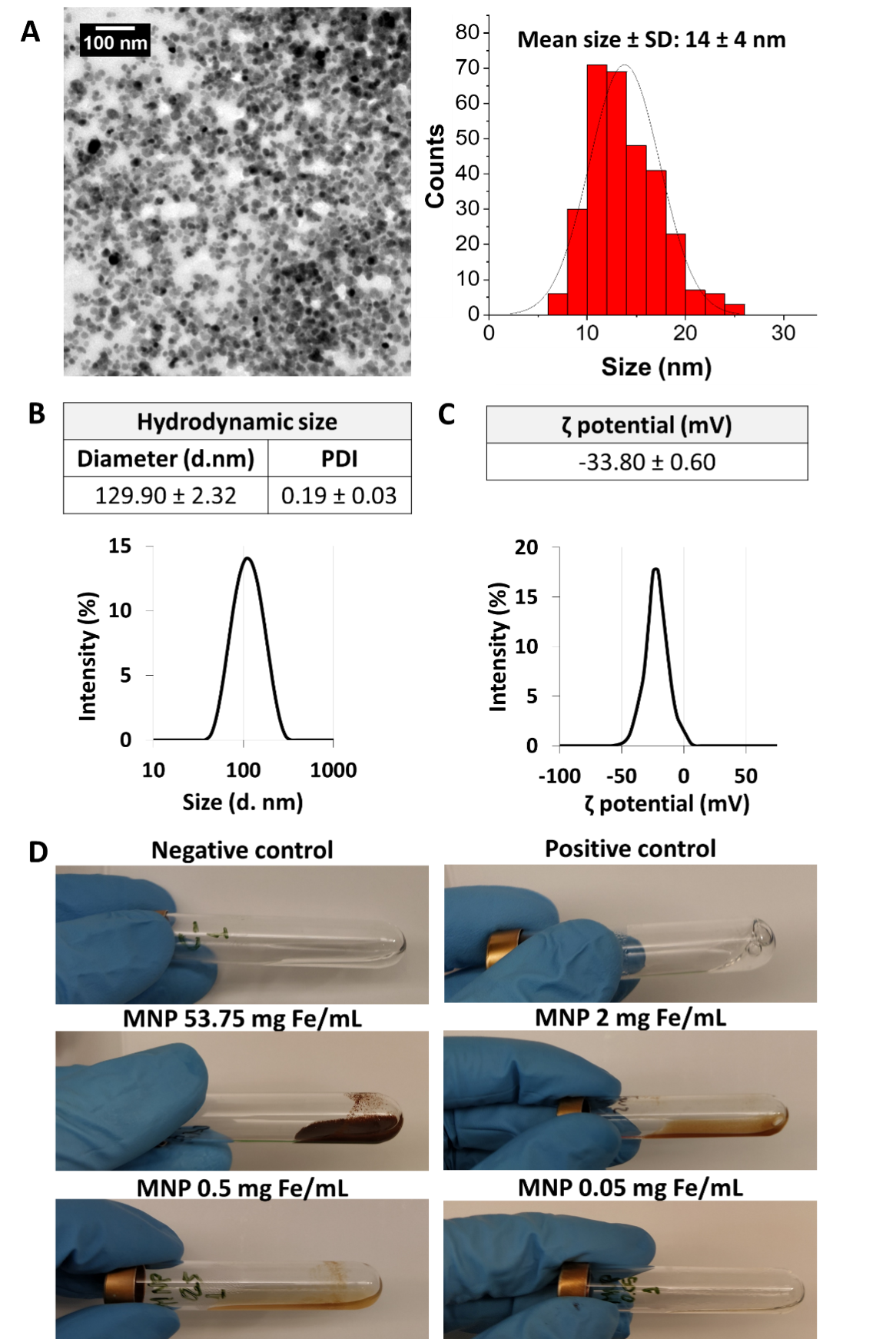


**Figure S1.-** Characterization of MNP. (A) TEM micrographs (left) and size distributions (right) of MNP. (B) Hydrodynamic size in terms of Z-average and polydispersity index (mean ± SD, n=3) and representative hydrodynamic size distribution in terms of intensity percentage. (C) ζ potential mean value ± SD (n=3) in water pH 7 and representative ζ potential distribution. (D) Gel-Clot assay test for the detection of endotoxins. The formation of a gel indicates the presence of endotoxins (positive control, 0.25 EU·mL^-1^).


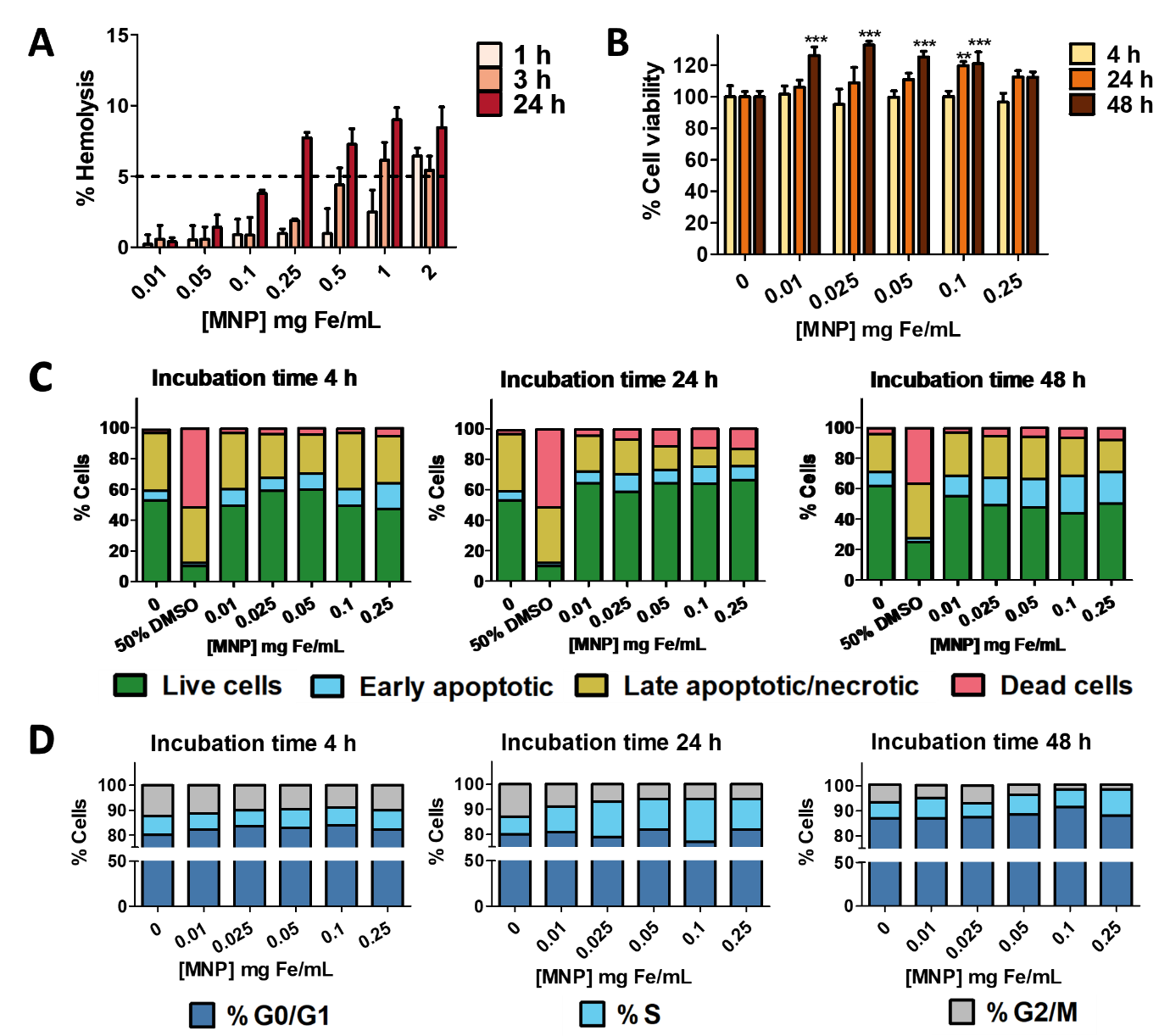


**Figure S2**.-*In vitro* biocompatibility studies with MNP. (A) Evaluation of the hemolysis activity of MNP in mice red blood cells after 1, 3 and 24 h of incubation. Data represent mean ± SD (n=3). (B) Evaluation of MNP toxicity in BMDC at different concentrations by Alamar Blue after 4, 24 and 48 h of incubation. Data represent mean ± SD (n=6). Statistical analysis was performed with One Way ANOVA test (untreated *vs* treated cells). ** p < 0.01, *** p < 0.001. (C) Necrosis/apoptosis analysis after incubation of MNP at different concentrations and incubation times in BMDC by Annexin V/7-ADD assay. (D) Cell cycle analysis of BMDC after incubation with MNP for 4, 24 and 48 h by PI staining.


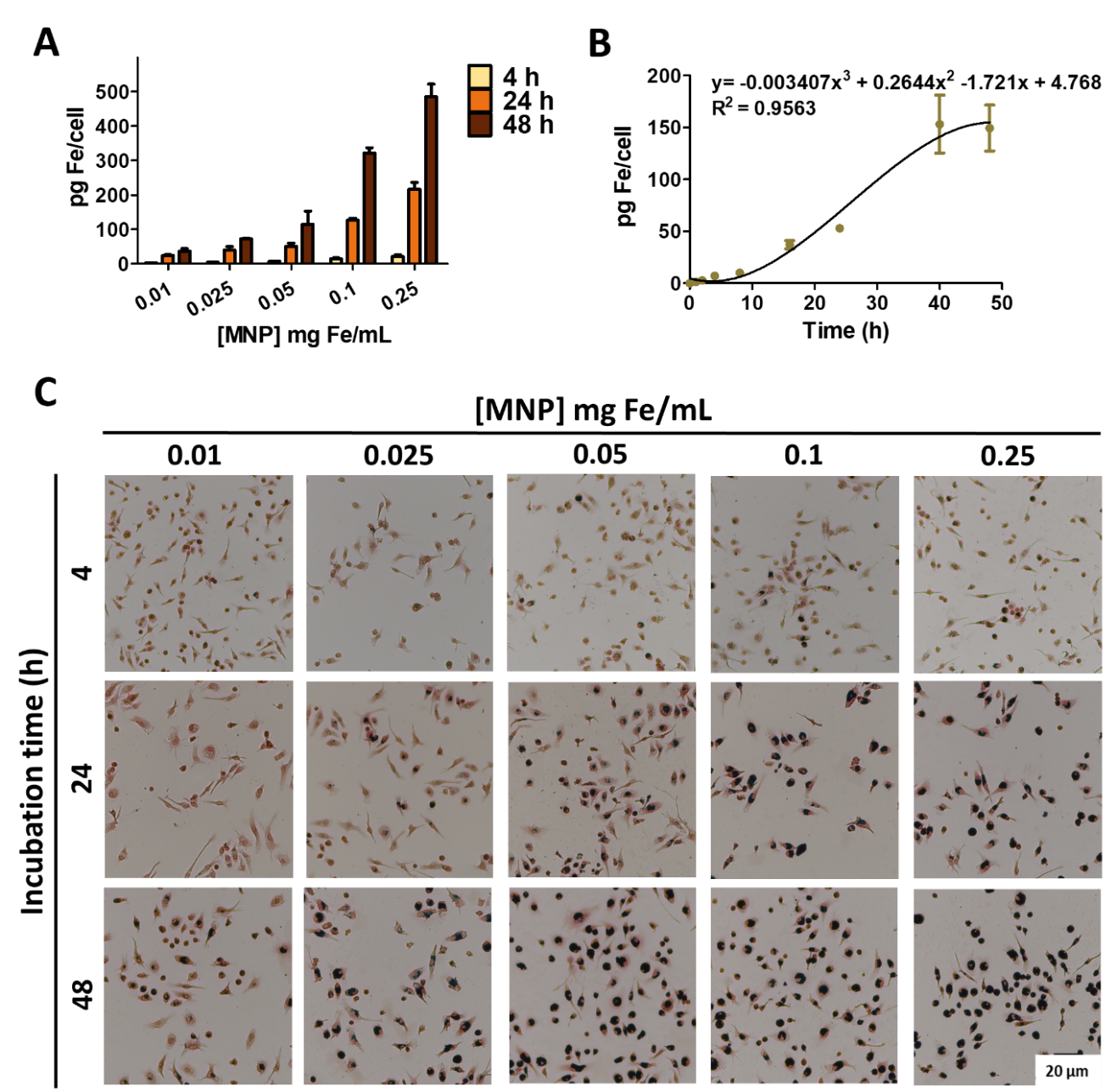


**Figure S3**.- Evaluation of MNP internalization in BMDC. (A) Quantification of iron in BMDC incubated with MNP by ferrozine assay. Data represent mean ± SD (n=3). (B) Kinetics of internalization of MNP in BMDC by quantification of iron by ferrozine assay. Data represent mean ± SD (n=3). (C) Prussian Blue staining photos of BMDC incubated with MNP after 4, 24 and 48 h of incubation in BMDC.


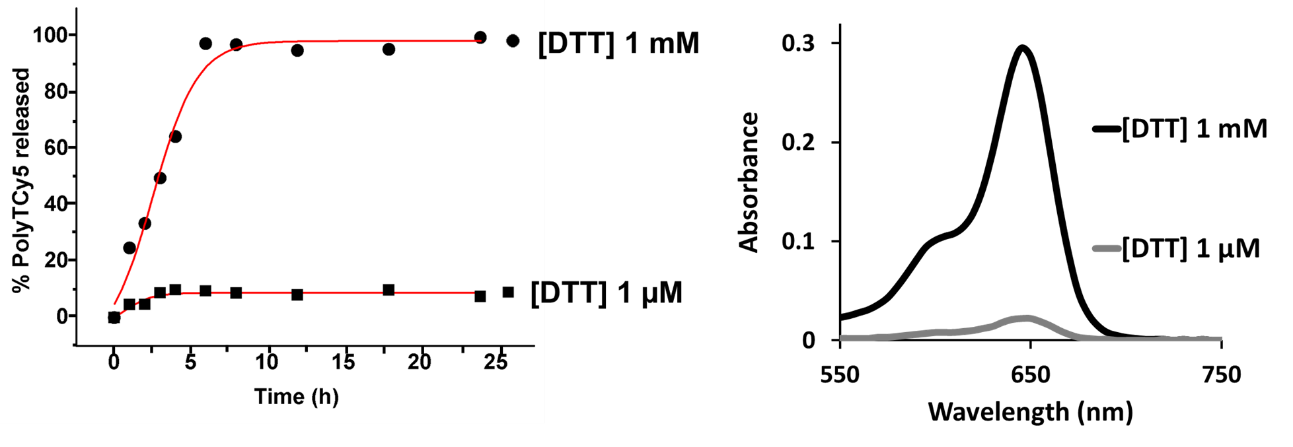


**Figure S4**.-.*In vitro* release studies with MNP-PolyTCy5 incubated with 1 mM or 1 µM DTT. Percentage of polyTCy5 released at different time points (left) and absorbance spectra of the released polyTCy5 after 24 h of incubation (right). (B) Confocal images of BMDC untreated and incubated with polyTCy5, MNP or MNP-PolyTCy5 after 4, 24 and 48 h of incubation (0.05 mg Fe·mL^-1^, 0.125 µM PolyTCy5). Phalloidin: green; DAPI: blue; Cy5: red.


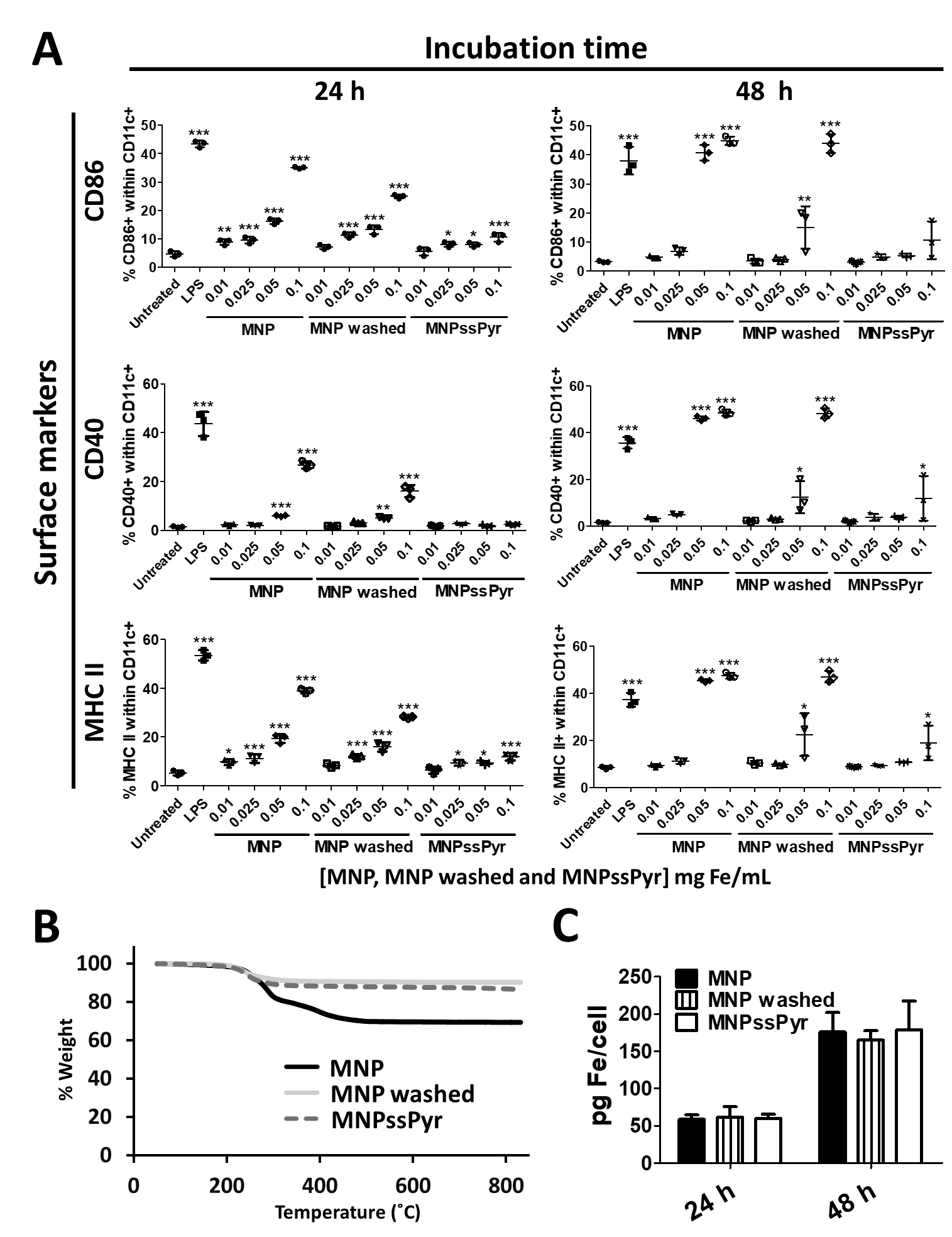


**Figure S5**.- Studies with MNP (carboxymethyldextran coated γ-Fe_2_O_3_), MNPssPyr (MNP functionalized with 2-pyridinethione groups via disulfide bonds) and MNP washed (MNP that were washed by the same cycles of centrifugation and redispersion than during the functionalization process of MNPssPyr). (A) Evaluation of maturation surface markers CD86, CD40 and MHCII expression in CD11c+ BMDC treated with MNP, MNP washed and MNPssPyr after 24 h and 48 h of incubation at different concentrations. Statistical analysis was performed with One Way ANOVA test (each condition *vs* untreated). * p < 0.05, ** p < 0.01, *** p < 0.001. (B) TGA analysis of MNP. MNP washed and MNPssPyr. (C) Quantification of iron in BMDC after 24 and 48 h of incubation with MNP, MNP washed and MNPssPyr (0.05 mg Fe·mL^-1^). Data represent mean ± SD (n=3).

**
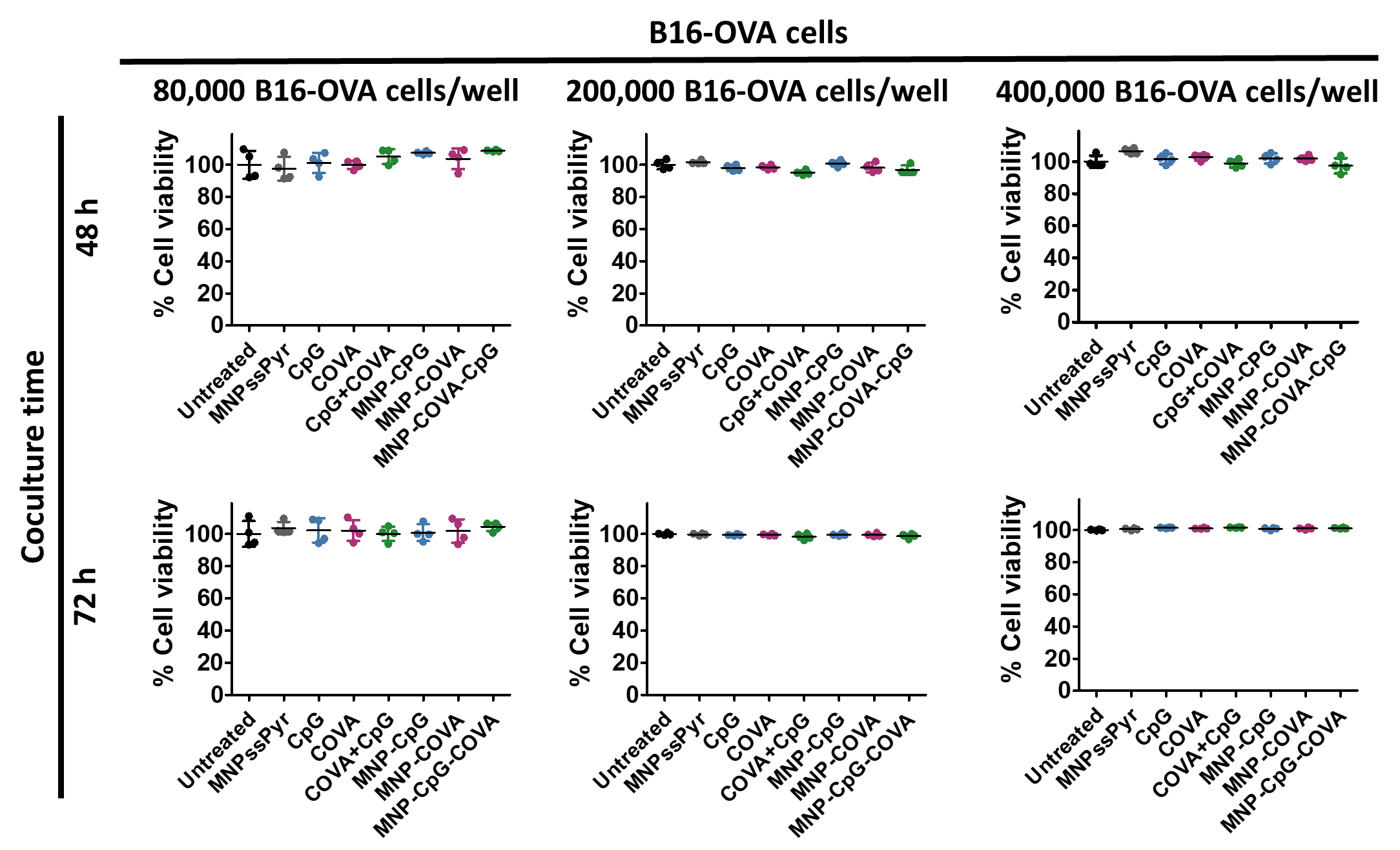
**

**Figure S6**.- *In vitro* tumor C57BL/6 CD8+ T cells killing assay. Detection of live and dead B16-OVA cells previously stained with CFSE. Data is represented as percentage of live cells compared to the untreated control (mean ± SD, n=4). BMDC were treated for 6 h with the corresponding treatments (0.05 mg Fe·mL^-1^; [CpG] 0.125 μM,[COVA] 0.125 μM), coincubated for 48 h with C57BL/6 CD8+ T cells and then cocultured for 48 or 72 h with different number of B16-OVA cells (initial BMDC: B16-OVA ratios 1:2, 1:5 and 1:10).


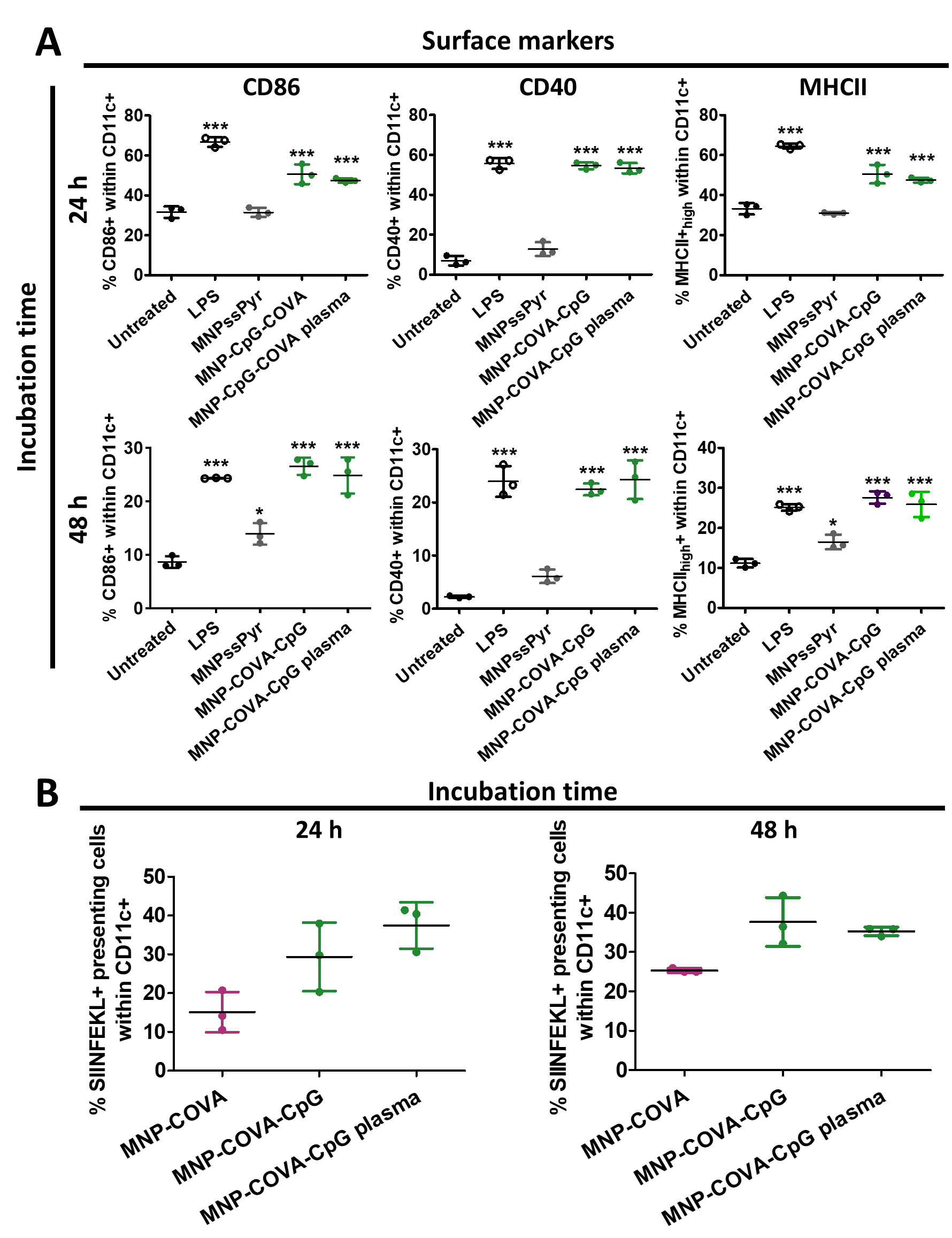


**Figure S7**.-*In vitro* studies of the effects of MNP-CpG-COVA in BMDC obtained from mice plasma. Conditions tested: [LPS] 5 ng/mL, [MNP] 0.05 mg Fe·mL^-1^, [CpG] 0.125 µM, [COVA] 0.125 µM. (A). Evaluation of maturation surface markers CD86, CD40 and MHCII expression in CD11c+ BMDC after 24 and 48 h of incubation with the corresponding treatments. Data represent mean ± SD (n=3). For statistical analysis One way ANOVA was performed (untreated *vs* each treatment). * p < 0.05, ** p < 0.01, *** p < 0.001. (B) Percentage of CD11c+ BMDC that present OVA_257-264_ (SIINFEKL) bound to MHCI after 24 and 48 h of incubation with the corresponding treatments. Data represent mean ± SD (n=3).

**
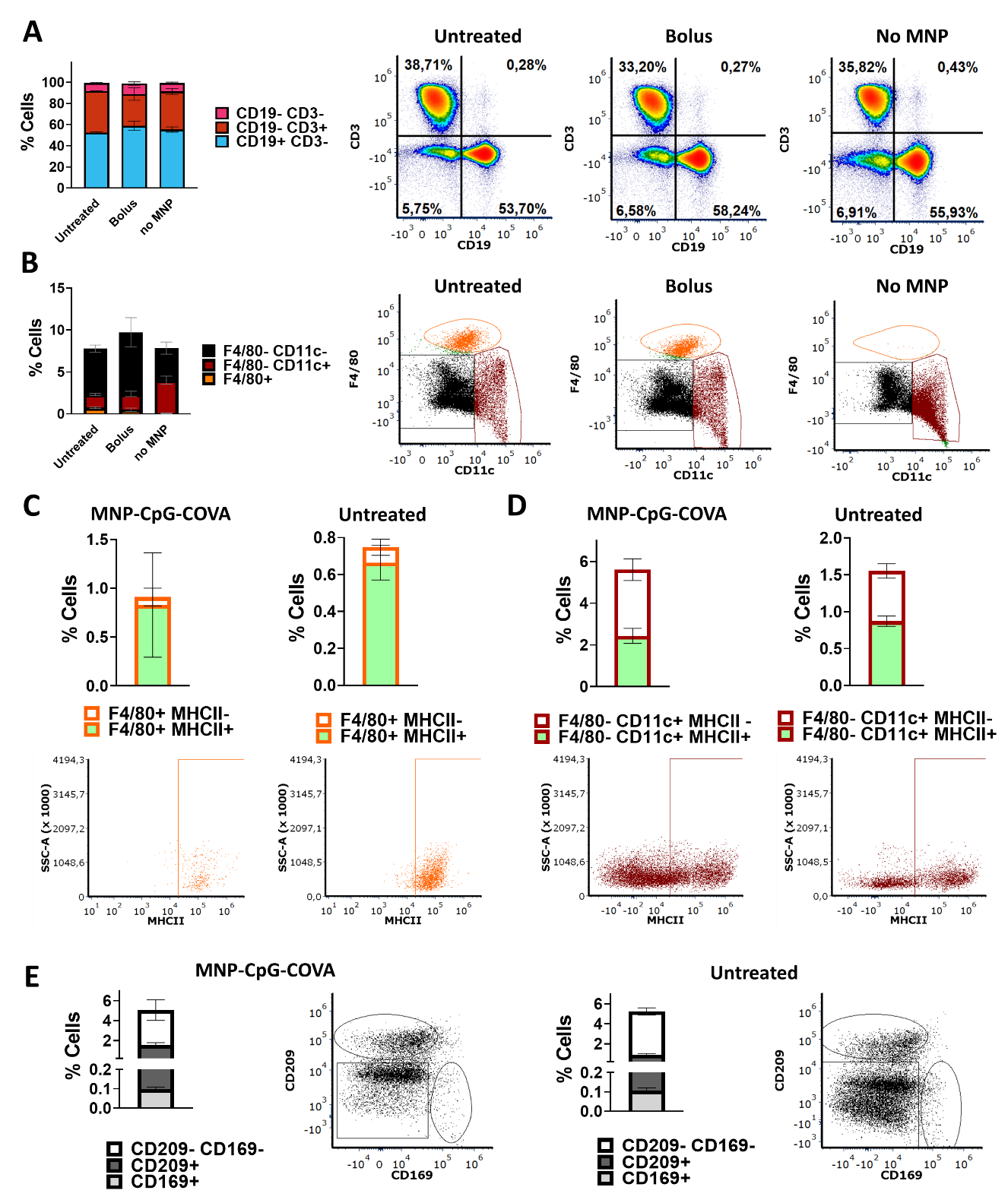
**

**Figure S8**.- Identification of spleen cells populations according to cell surface markers expression. (A) Percentage of spleen cells as a function of CD19 and CD3 markers expression (left) and representative quadrants of cells populations (right). (B) Percentage of spleen cells as a function of F4/80 and CD11c expression within CD19- CD3- population (left) and representative quadrants of cells populations (right). (C) Percentage of spleen cells as a function of MHCII expression within F4/80+ CD19- CD3- population (top) and representative quadrants of cells populations (bottom). (D) Percentage of spleen cells as a function of MHCII expression within F4/80- CD11c+ CD19- CD3- population (top) and representative quadrants of cells populations (bottom). (E) Percentage of spleen cells as a function of CD209 and CD169 expression within F4/80- CD11c- CD19- CD3- population (left) and representative quadrants of cells populations (right).


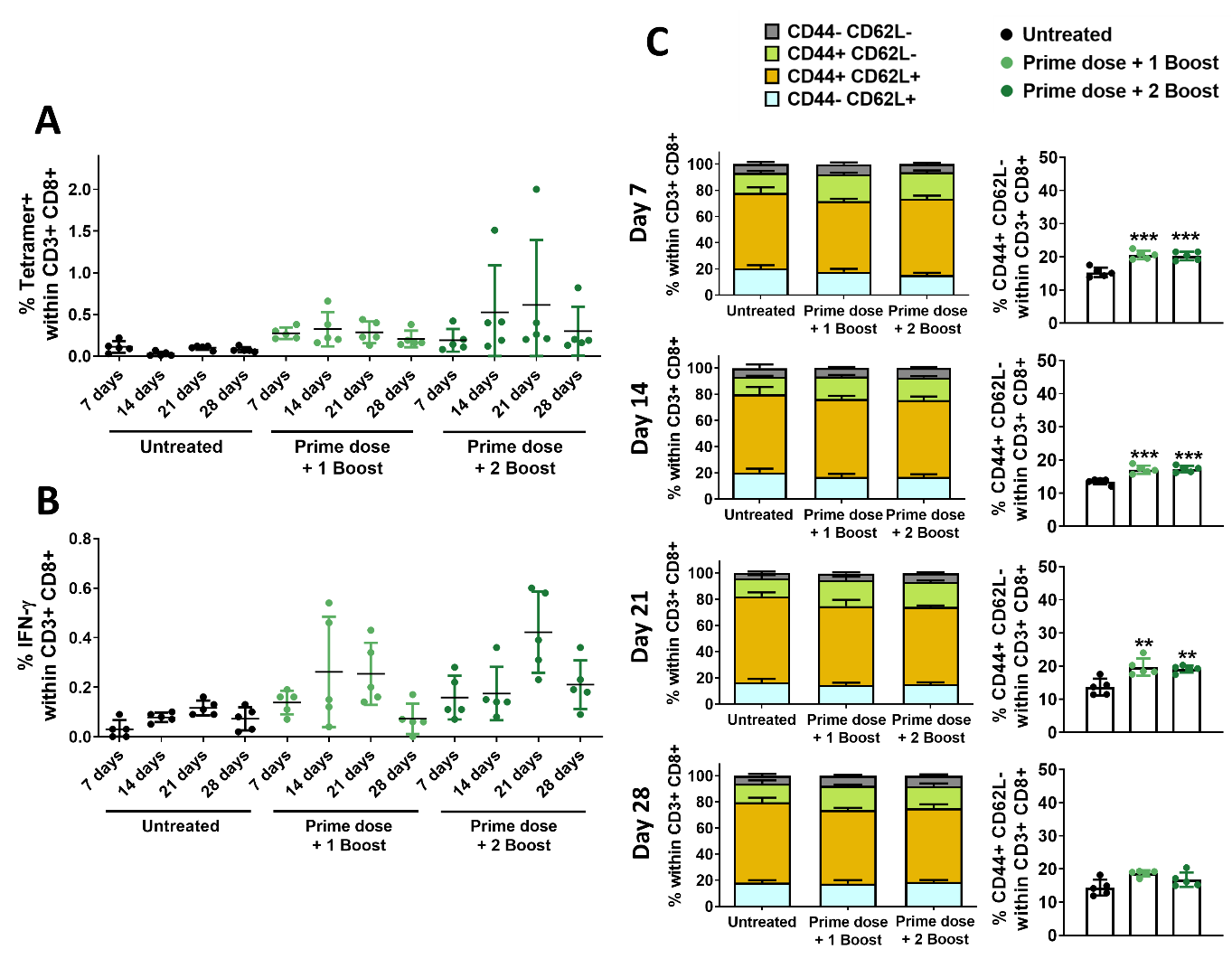


**Figure S9**.- *In vivo* evaluation of MNP-CpG-COVA (0.5 mg Fe, 12.5 nmol CpG, 12.5 nmol COVA) effects in the immune response in C57BL/6 mice. (A) Percentage of tetramer+ CD8+ T cells. (B) Percentage of IFN-γ+ CD8+ T cells. (C) Division of CD8+ T cells according to CD44 and CD62L expression (left) and percentage of CD8+ effector T cells (CD44+ CD62L-) (right). Statistical analysis was performed with One Way ANOVA test. * p < 0.05, ** p < 0.001, *** p < 0.001.


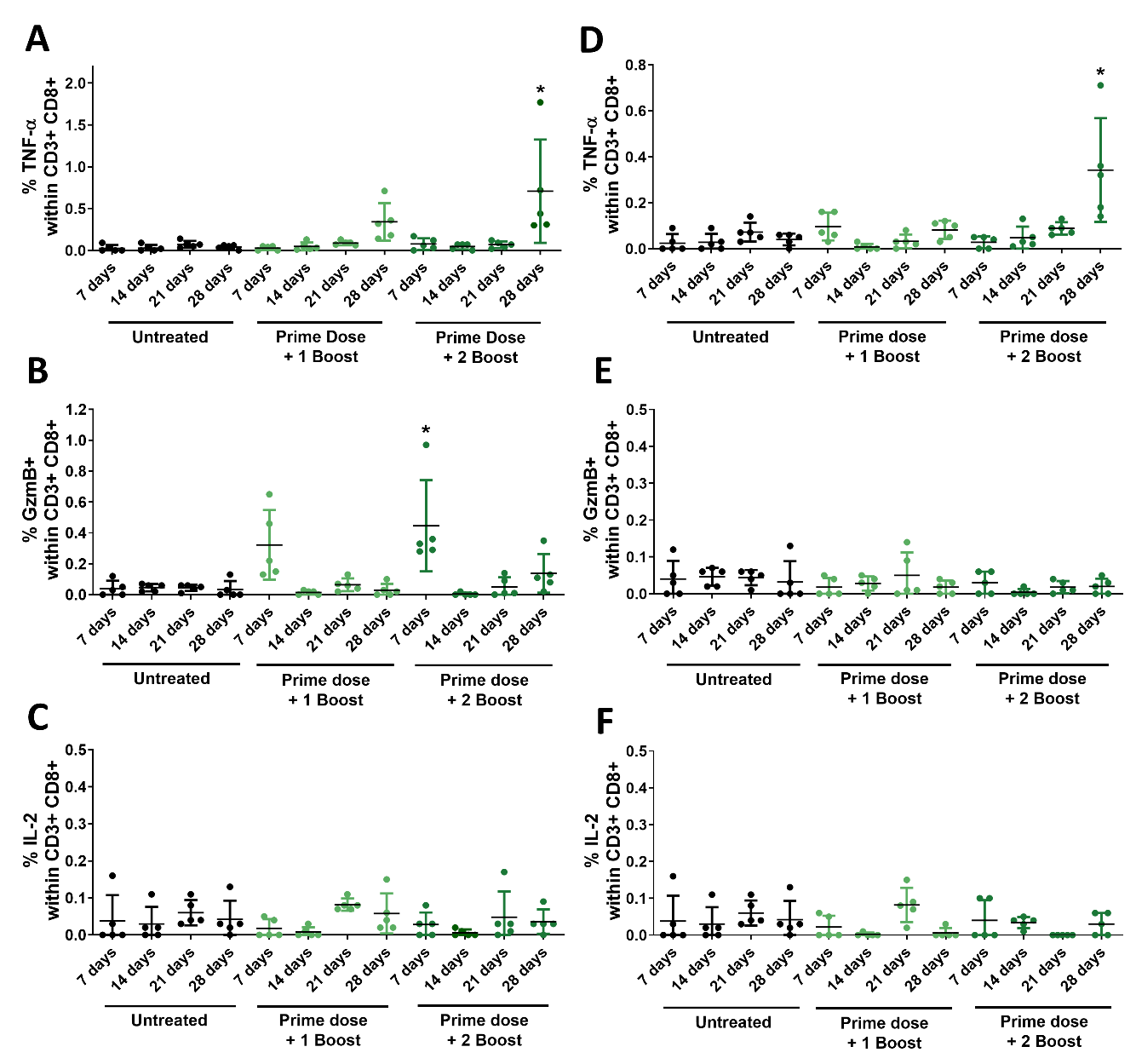


**Figure S10**.- *In vivo* evaluation of MNP-CpG-COVA effects in the immune response in C57BL/6 mice. Vaccination with MNP-CpG-COVA 5 mg Fe, 125 nmol CpG, 125 nmol COVA (A-C) and vaccination with MNP-CpG-COVA 0.5 mg Fe, 12.5 nmol CpG, 12.5 nmol COVA (D-F). (A and D) Percentage of TNF-α+ CD8+ T cells. (B and E) Percentage of GzmB+ CD8+ T cells. (C and F) Percentage of IL-2+ CD8+ T cells. Statistical analysis was performed with One Way ANOVA test. * p < 0.05.


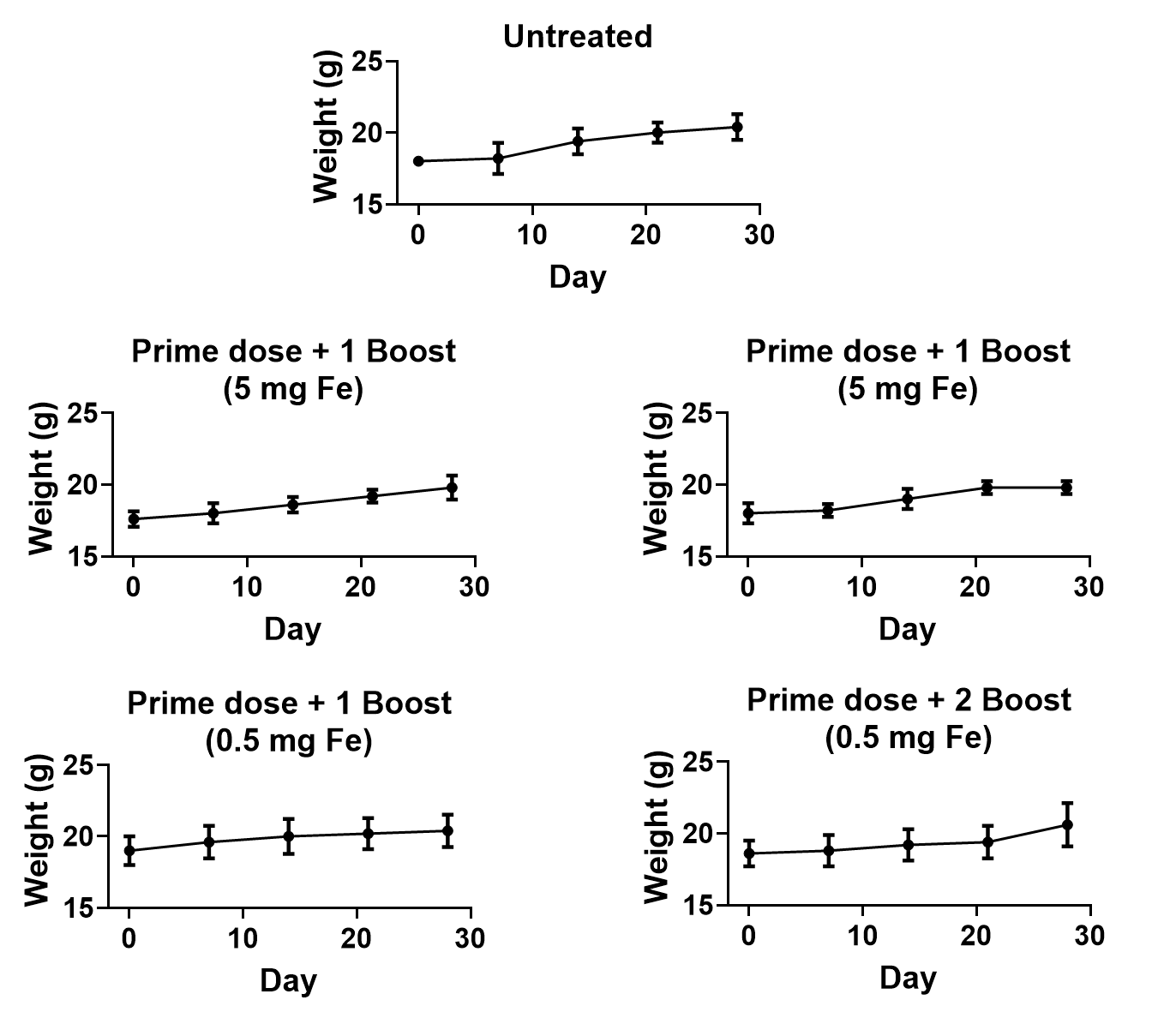


**Figure S11**.- Body-weight changes in mice according to their vaccination scheme. Data is represented as mean ± SD (n=5).


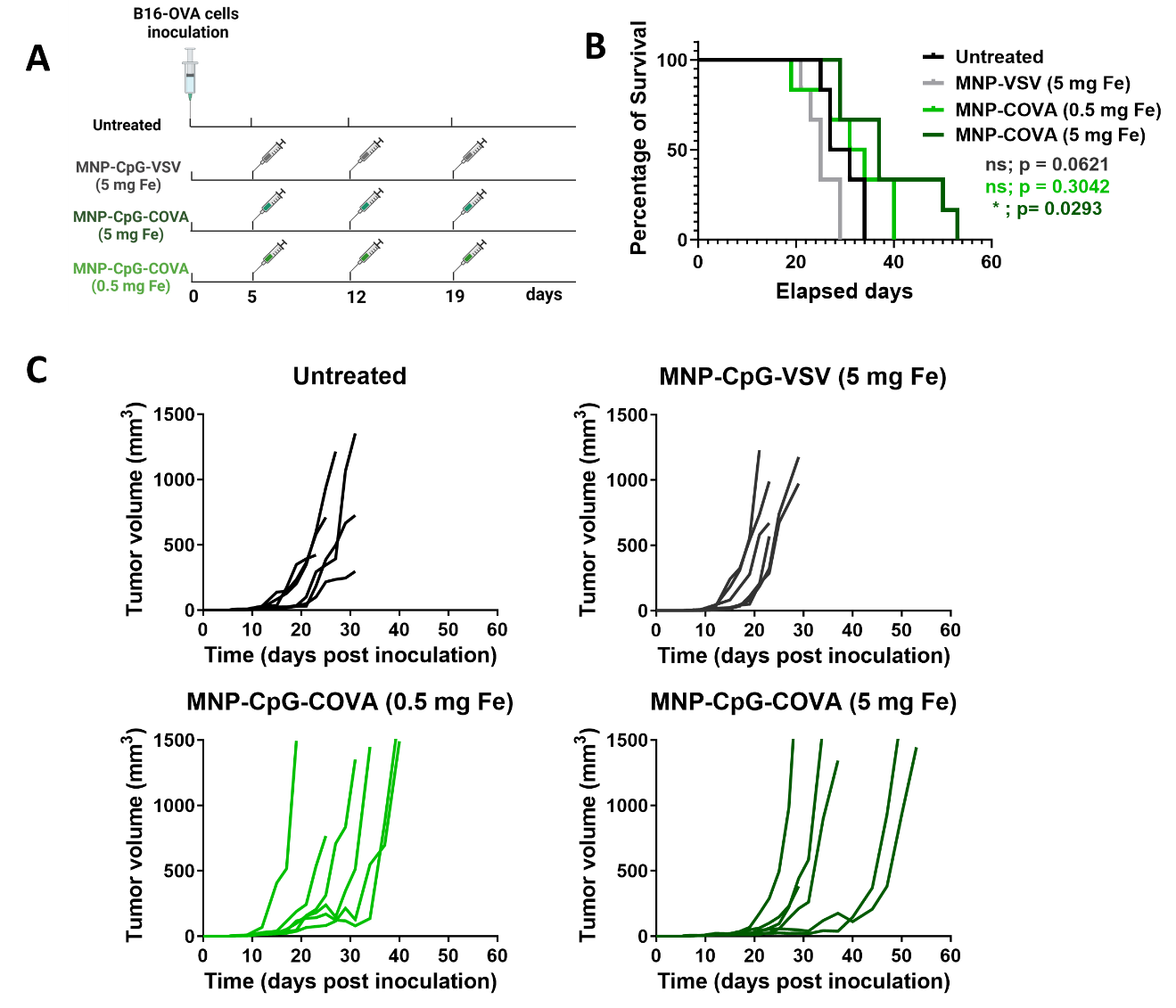


**Fig. S12.-** (A) Schematic timeline representation of B16-OVA cells inoculation (1 x 10^5^ cells/animal) and vaccination timepoints (n=6 per group). (B) Overall survival of mice bearing established B16-OVA tumors.Log-rank (Mantel-Cox) test was employed to compare the survival curves (untreated vs. each condition).(C)Tumor growth of mice bearing established B16-OVA tumors. Data depict individual tumor growth. Conditions tested: MNP-CpG-COVA 5 mg Fe, 125 nmol CpG, 125 nmol COVA; MNP-CpG-COVA 0.5 mg Fe, 12.5 nmol CpG, 12.5 nmol COVA, and MNP-CpG-VSV 5 mg Fe, 125 nmol CpG.

**Suplementary Information about Experimental Section**

**Synthesis and characterization of magnetic nanoparticles (MNP)**

Briefly, to an aqueous solution (425 mL) of FeCl_2_·4H_2_O (Sigma Aldrich; 0.126 M) and FeCl_3_·6H_2_O (Sigma Aldrich; 0.212 M), NH_4_OH 25 % (Sigma Aldrich; 75 mL, 0.1 mL·s^-1^) was added under vigorous stirring at room temperature for 5 min. Later, the reaction was heated (90 ºC, 3 h), washed three times with water by magnetic decantation and stirred overnight at room temperature. The obtained precipitate was isolated by magnetic decantation, HNO_3_ was added (Sigma Aldrich; 300 mL, 2 M) and the mixture stirred for 15 min. Then, HNO_3_ 65 % was removed by magnetic decantation, and Fe(NO_3_)_3_·9H_2_O (Sigma Aldrich; 75 mL, 1M) and distilled water (130 mL) were added and the mixture was boiled for 30 min. The supernatant was removed by magnetic decantation, HNO_3_ (300 mL, 2M) was added and the mixture was stirred for 15 min. The γ-Fe_2_O_3_ cores obtained were washed with water and concentrated in a rotary evaporator. For the coating, the γ-Fe_2_O_3_ cores (800 mg, 2 mL miliQ water) were mixed with carboxymethyldextran (Sigma Aldrich; 800 mg, 10 mL miliQ water) and HNO_3_ 65 % was added to adjust the pH at 3. The mixture was sonicated for 10 h under refrigeration (20- 45 ºC). The obtained coated nanoparticles (MNP) were centrifuged using amicon ultra centrifugal filters and the pH was adjusted to 7.

**Hemocompatibility studies with mice RBCs**

Fresh mice RBC were obtained from the blood of C57BL/6 mice. Briefly, the blood was washed by three cycles of centrifugation (835 g x 4 min) and redispersion with a 150 mM NaCl solution (Sigma Aldrich) to remove the plasma and protective reagents from the collector tubes. MNP dispersions at different concentrations were prepared in 200 µL PBS (n=3), 2 x 10^6^ RBC were added and the mixtures were incubated for 1, 3 and 24 h. Then, all samples were centrifuged (1485 g x 4 min), the pellet with the intact RBC was discarded and the supernatant was centrifuged again (4000 g x 10 min) to force the precipitation of all possible MNP present in the solution. Then, the absorbance of the supernatants was measured in a Synergy Neo2 Plate Reader (BioTek Instruments) to detect the hemoglobin (λ_540 nm_). The 100 % of hemolysis (positive control) was defined by incubating RBC in miliQ water, while the 0 % of hemolysis (negative control) corresponded to RBC incubated in PBS without nanoparticles. The % of hemolysis was calculated according to the equation S1:

$\% Hemolysis=\frac{{Abs 540 nm}_{sample}-{Abs 540 nm}_{negative control}}{{Abs 540 nm}_{positive control}-{Abs 540 nm}_{negative control}}$ (S1)

The hemolytic grade of the nanoparticles was determined after 3 h of incubation according to the instructions of the *ASTM E2524-08(2013)*: 0-2 % non-hemolytic, 2-5 % slightly hemolytic, > 5 % hemolytic.

**Ferrozine assay**

The cell lysates previously digested with NaOH 10 mM were mixed with 100 µL HCl 10 mM and 100 µL of iron releasing reagent (1.4 M HCl and 4.5% w/w KmnO_4_ in water, all products from Sigma Aldrich). The mixtures were heated at 60 ºC for 2 h and then cooled down to room temperature. Then, 30 µL of iron detection reagent were added (6.5 mM ferrozine, 6.5 mM neocuproine, 2.5 M ammonium acetate, and 1 M ascorbic acid in miliQ water; Sigma Aldrich). After 30 min of incubation, the absorbance at 565 nm was measured in a Synergy Neo2 Plate Reader (BioTek Instruments). The same procedure was employed to do a calibration line with MNP at known concentrations.

**Prussian Blue staining**

For the staining, a solution of 2 % HCl and 2 % potassium ferrocyanide trihydrate (Sigma Aldrich) was added and incubated for 15 min. Later, cells were washed with PBS, dried and counterstained with a solution of 0.5 % neutral red (Sigma Aldrich) in miliQ water for 3 min. The preparations were visualized in a Zeiss Axiophot Microscope and the images were processed using Fiji software (ImageJ).

**Evaluation of BMDC activation state**

For the gating strategy, CD11c+ viable cells were selected to evaluate the expression of CD86+, MHCII+, and CD40+ populations. In all cases, the results were expressed as % marker expression within CD11c+ population, as described in Fig. S13.


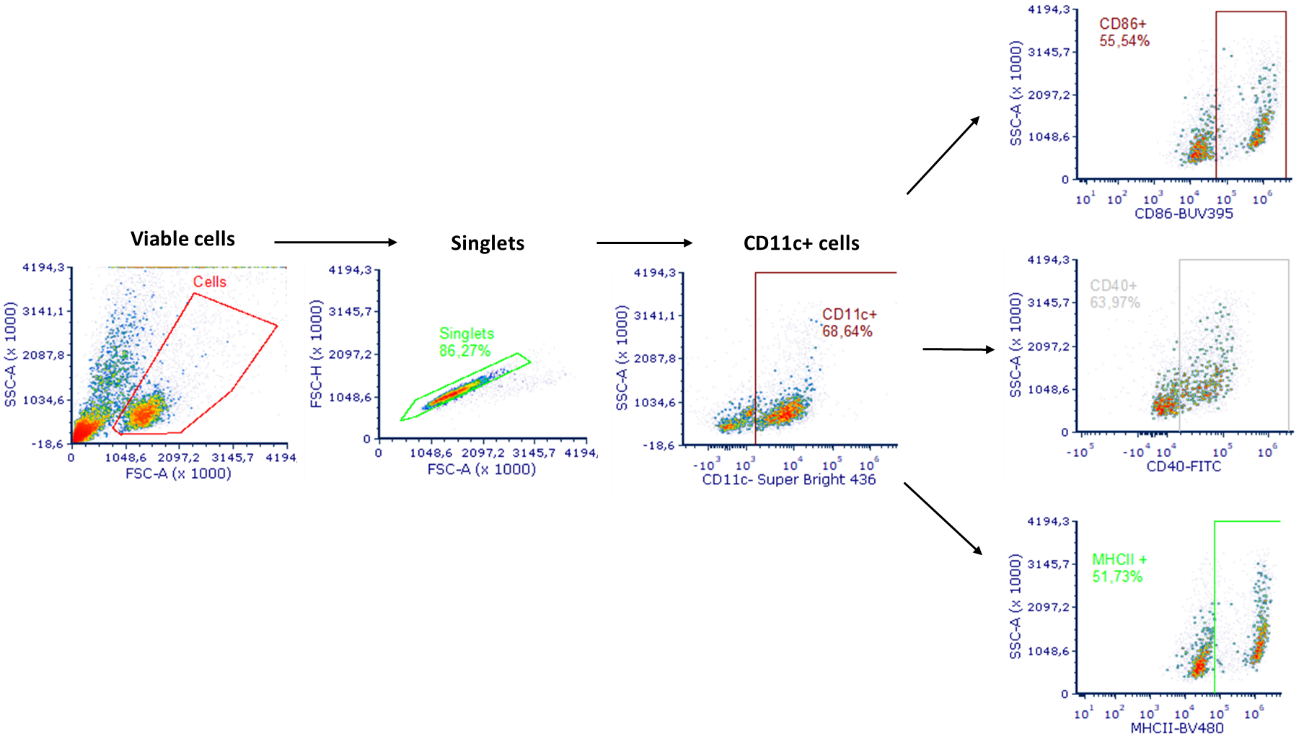


**Figure S13.** A representative gating strategy illustrating dendritic cells being subgated to the level of CD86+, MHCII+ and CD40+ cells within CD11c+ population.

**CD8+ T cell expansion study**

For the gating strategy, live CD3+ cells were gated and the CFSE dilution was analyzed as a marker for cell division, as described in Fig. S14.


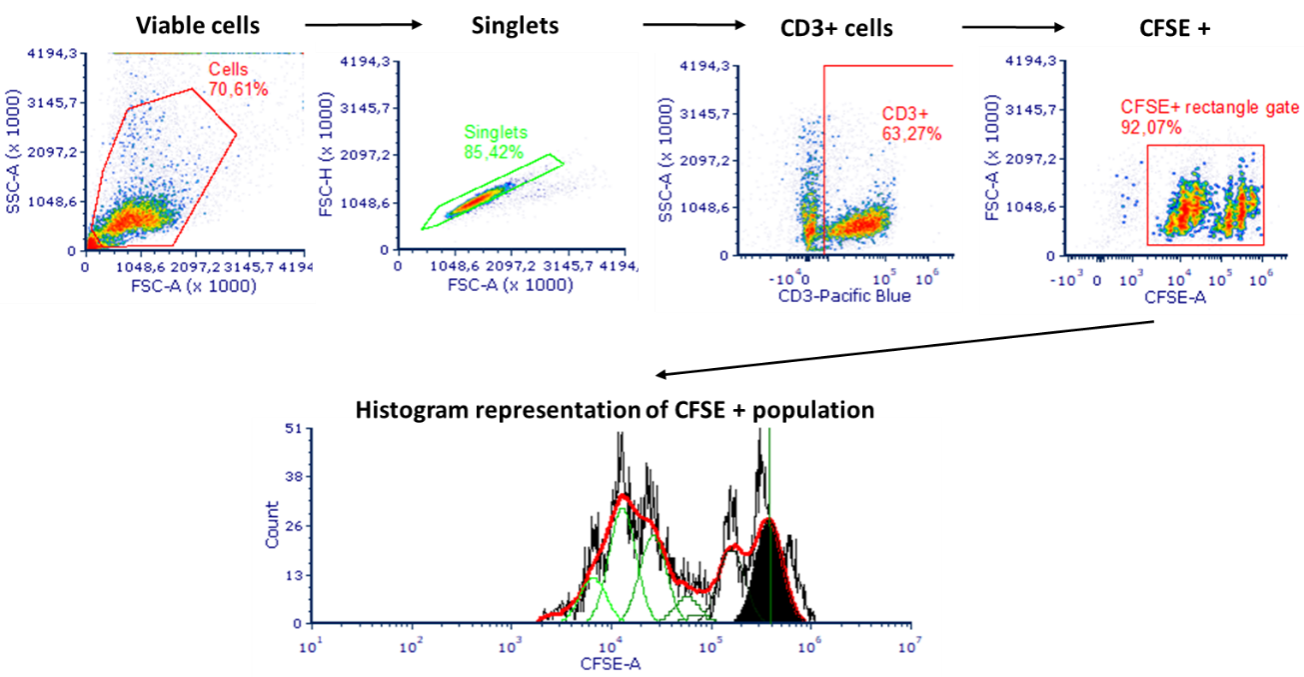


**Figure S14.** A representative gating strategy illustrating CFSE+ T cells being subgated to evaluate their proliferation.

**Study of the immune response and biodistribution of MNP-CpG-COVA in vivo**

For the gating strategy, live CD3+ CD8+ cells were gated and several parameters were analyzed,as described in Fig. S15.

**
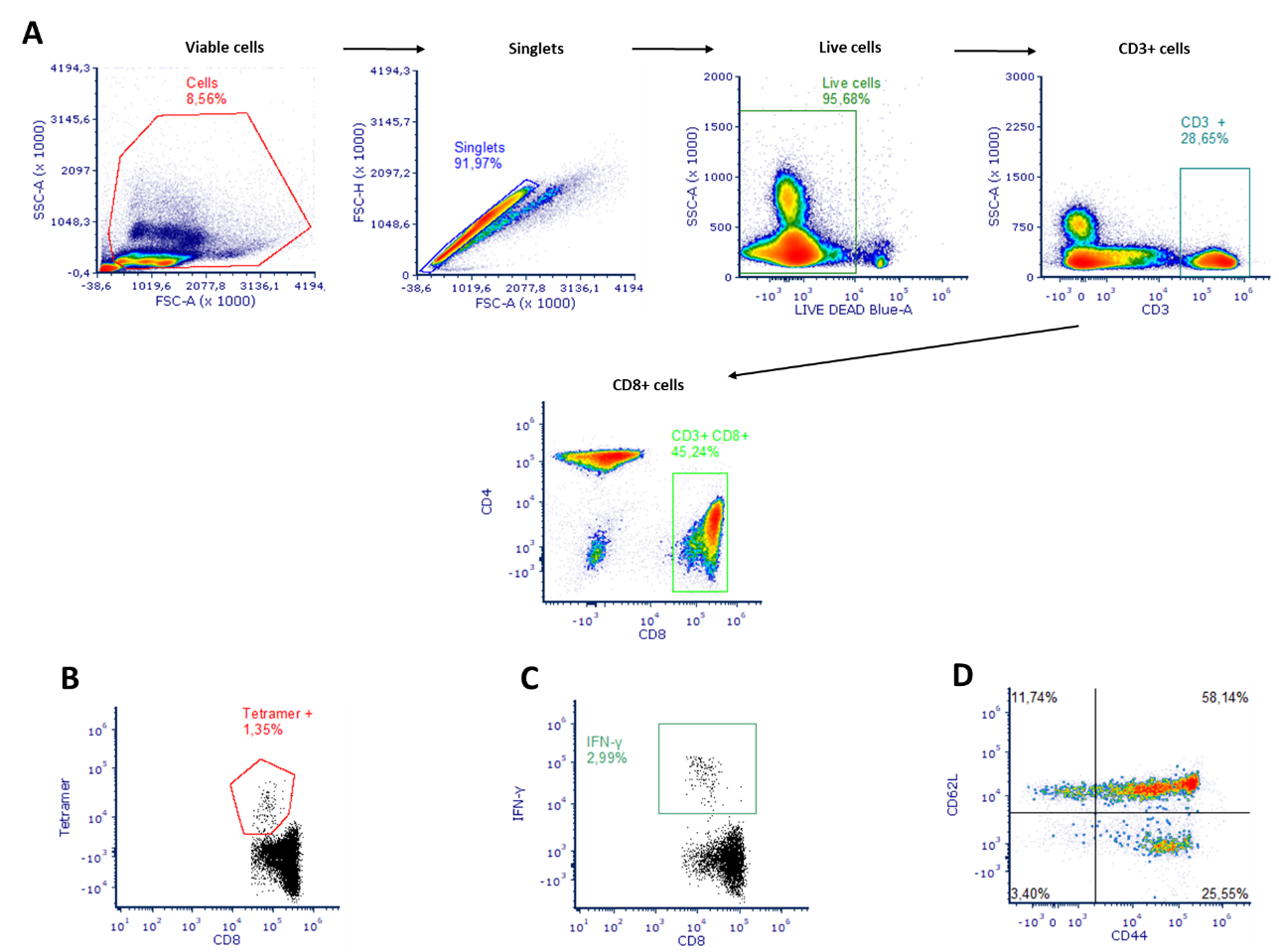
**

**Figure S15.** (A) A representative gating strategy illustrating PBMCs being subgated to identify the CD3+ CD8+ cells. (A) A representative gating of Tetramer + cells within CD3+ CD8+ population. (C) A representative gating of IFN-γ + cells within CD3+ CD8+ population. (C) Schematic for gating CD44- CD62L-, CD44+ CD62L-, CD44- CD62L+, CD44+ CD62L+ cells within CD3+ CD8+ population.
